# OsteoBLAST: Computational Routine of Global Molecular Analysis Applied to Biomaterials Development

**DOI:** 10.3389/fbioe.2020.565901

**Published:** 2020-10-08

**Authors:** Marcel Rodrigues Ferreira, Renato Milani, Elidiane C. Rangel, Maikel Peppelenbosch, Willian Zambuzzi

**Affiliations:** ^1^Department of Chemistry and Biochemistry, Institute of Biosciences, São Paulo State University (UNESP), São Paulo, Brazil; ^2^Bioquímica e Biologia Tecidual, Biology Institute, Universidade de Campinas (UNICAMP), São Paulo, Brazil; ^3^Institute of Science and Technology, São Paulo State University (UNESP), São Paulo, Brazil; ^4^Department of Gastroenterology and Hepatology, Erasmus MC, University Medical Center Rotterdam, Rotterdam, Netherlands

**Keywords:** biomaterials, bone healing, bioinformatics, alternative methods, analysis

## Abstract

For bone purposes, surface modifications are a common trend in biomaterials research aiming to reduce the time necessary for osteointegration, culminating in faster recovery of patients. In this scenario, analysis of intracellular signaling pathways have emerged as an important and reliable strategy to predict biological responses from *in vitro* approaches. We have combined global analysis of intracellular protein phosphorylation, systems biology and bioinformatics into an early biomaterial analysis routine called OsteoBLAST. We employed the routine as follows: the PamChip tyrosine kinase assay was applied to mesenchymal stem cells grown on three distinct titanium surfaces: machined, dual acid-etched and nanoHA. Then, OsteoBLAST was able to identify the most reliable spots to further obtain the differential kinome profile and finally to allow a comparison among the different surfaces. Thereafter, NetworKIN, STRING, and Cytoscape were used to build and analyze a supramolecular protein-protein interaction network, and DAVID tools identified biological signatures in the differential kinome for each surface.

## Introduction

Osseous injuries are one of the most common problems in the dental and medical fields. Titanium implants are widely used in bone regeneration as a bone substitute material (Henkel et al., 2013; Ko et al., 2017); preliminarily based on their physical and chemical properties. Unquestionably, a decisive factor for the success of these implants is their surface (Bertazzo et al., 2009, 2010; Ribeiro et al., 2015; Fernandes et al., 2018; Feltran et al., 2019), which will be first in contact with the host’s blood. It is known that physicochemical modifications in implant surfaces affect cellular behavior and directly impact osteointegration (Gemini-Piperni et al., 2014a,b; Zambuzzi et al., 2014; Bezerra et al., 2017; Fernandes et al., 2018; Machado et al., 2019). Despite the medical, social and economic relevance of such biomaterials, there have been few advances in tools to classify and rank them, which results in universities and companies using traditional testing, often with animal experimentation as a model.

Regardless, the 3Rs concept introduced by Russel and Burch in 1959 has ethically guided research involving animal experimentation over the world (Eder et al., 2006). In different areas, researchers are urged to develop alternatives under 3Rs guidance and some methodologies deserve attention such as 3D cultures, *in vitro* cell cultures, mimetic tissues, computational tools among others (Cook et al., 2015; Gordon, 2015; Zhu, 2016). In bone tissue engineering some steps are being taken in the direction of the 3Rs (Richards et al., 2007; Henkel et al., 2013), but in regard to the production of dental, and medical biomaterials, there is still a dearth of procedures that minimize animal experimentation. In this sense, the study of cellular adhesion on the implant surface by alternative methods comes up as a promising possibility (Zambuzzi et al., 2011a, 2014).

Adequate adhesion of osteoblasts on the surface of the implant is within the first stage for osteointegration and is controlled by signal transduction mechanisms responding to various stimuli, such as the chemical properties of material surfaces (Bertazzo et al., 2009, 2010; Zambuzzi et al., 2011b, 2014). These mechanisms are finely regulated by cascades of protein phosphorylation leading to a cell response. Phosphorylation is a post-translational and covalent modification of proteins, which concludes with significant regulations of various cellular processes such as migration, adhesion, proliferation, differentiation, among others (Milani et al., 2010; Marumoto et al., 2017; da Costa Fernandes et al., 2018; Kang et al., 2018; Baroncelli et al., 2019). In this context, it is important to note that kinases catalyze phosphorylation in a specific site-dependent manner, and are responsible, together with phosphatases, for controlling signal transduction pathways. The human genome contains 518 putative kinase-encoding genes, the set of which is known as the kinome (Manning, 2002). Massive studies of this set of enzymes have been performed over the last years and recent advances in Bioinformatics and Molecular Biology have allowed for a broader and more efficient analysis of cellular metabolism and signaling pathways. However, biologically, it is more important to focus on enzymatic activity rather than metabolite concentration or gene expression, which during the cell adhesion process on biomaterials appears as a promising alternative to predict the success of an implant (Zambuzzi et al., 2014).

A valuable tool in this type of analysis is a microarray of peptides. Using a single chip it is possible to probe the phosphorylation status of hundreds of enzymes. The Tyrosine Kinase PamChip^®^ array is a 144 peptide chip, with each representing sites of known phosphorylation (Dussaq and Anderson, 2016; Baharani et al., 2017; Baroncelli et al., 2019), Hence, it is a key in the search for the differential kinome activity of cell-biomaterial interactions.

We were, therefore, prompted to develop an algorithm that, based on a peptide microarray assay, was able to distinguish the kinome activity of mesenchymal stem cells response to surfaces with different topographies and to compare this response to commonly used materials. Thus, this routine will serve as the basis for a database capable of assisting in the production of biomedical and medical devices.

## Materials and Methods

### Materials and Characterization

Three different surfaces were used in this study: machined (Maq), dual acid-etched (DAA) and nanoHA. The surface microstructure of the samples was evaluated by secondary electron micrographs collected in a JEOL JSM 6010LA microscope. The micrographs were acquired with 3 kV acceleration potential, Spot Size (SS) of 30 and 2500 X amplification. To avoid surface charging during inspections, a thin conductive layer was deposited on the surfaces by the sputtering of an Au-Pd alloy. The micrographs were acquired from the most representative region of each sample.

The elemental composition of the surfaces was determined by Dispersive Energy Spectroscopy using an X-ray detector (Dry SD Hyper EX-94410T1L11) coupled to a scanning electron microscope with a resolution of 129 to 133 eV for the Mn Kα line at 3000. For the analysis of elemental composition, beam acceleration voltage of 5 kV, Spot Size of 70 and magnification of 500 X were used. Area spectra as well as maps of the distribution of the elements on the surface of the samples were recorded.

Wettability of the samples was determined by the sessile drop method on a Ramé-Hart 100-00 goniometer. Droplets with 2 μL deionized water and diiodomethane were used as test liquids. The contact angle between the drop and the surface was measured ten times on each side of the drop. As three drops were deposited on the surface of each sample, 60 values of contact angle per sample were obtained. Surface energy was determined using the measured contact angle values for water and diiodomethane with the software provided by the equipment manufacturer.

### Cell Culture

Human bone marrow-derived MSCs were used in this study. Briefly, MSCs (5128 viable cells/cm^1^; PT-2501, Lonza, Walkersville, MD, United States) from a single donor at passage 7 were cultured in growth medium for 2 days (alpha-Mem phenol-red free (GIBCO, Paisley, United Kingdom), 10% fetal bovine serum).

### Tyrosine Kinase Activity Profiling Using PamChip Peptide Microarray

The experiment was performed according to previously described procedures in Sikkema et al. (2009). To check the effect of the titanium surfaces on MSC behavior, MSCs (28300 viable cells/cm^2^) were cultured on these surfaces in growth medium. After 4 h, cells were scraped in M-PER Mammalian protein extraction buffer (Thermo Scientific, Rockford, IL, United States) containing Halt phosphatase and protease inhibitors (Thermo Scientific), allowed to lyse at 4°C for 10 min and lysates were cleared by centrifugation at 14,000 *g* for 10 min. Supernatants were stored at –80°C until use. Cell lysates (5 μg of protein for all samples) were loaded on a PamChip tyrosine kinase microarray (PamGene International BV., Hertogenbosch, Netherlands). PamChip^®^ is a high-throughput and cost-effective peptide array that allows the study of kinome profile changes without *a priori* assumptions (Peppelenbosch, 2012). In the PamChip platform, cell lysates are continuously pumped past 144 consensus peptide-sequences spotted on a 3D porous microarray, and the phosphorylation of their specific target substrates by kinases present in the whole cell lysate is fluorescently detected, describing the entire tyrosine kinase activity profile within a single experiment (Diks et al., 2004; Lemeer et al., 2007; Sikkema et al., 2009). Phosphorylation of the 144 kinase substrates on the array was detected using FITC-labeled secondary antibody. After array washing, images were taken every 5 min to create real-time kinetics data. Signal intensities of the three technical replicates for each substrate were quantified using Bionavigator software (version 6.1.42.1, PamGene International BV). A complete list of phosphopeptides on PamChip is depicted in Supplementary Table S5. The internal positive control peptide ART_003_EAI(pY)AAPFAKKKXC was not considered for further analysis. Kinase reactions start at *t* = 640 s. Subsequently, kinase reactions for different peptides show markedly different kinetics. Most peptides act according to classical biochemical theory, with the derivative of the initial reaction speed approximating maximal velocity (Vmax) for phosphorylation of these peptides.

### OsteoBLAST Platform Analysis

OsteoBLAST algorithm were built in the programming environment R^2^. Spots with negative values were manually set to zero. Reliable spots were selected using two parameters, P1 = sd/A and P2 = A/M (sd = standard deviation; A = average; M = median). These two parameters were defined with three levels: High, Medium, and Low. The range of these two parameters are defined as: P1 < 20% and 80% < P2 < 120% – High. P1 < 50% and 70% < P2 < 140% – Medium. Values out of those ranges were considered Low. In order to obtain the highest possible number of spots with high reliability, parameters P1 and P2 have been combined in a new parameter here named SR, with minimum 1 and maximum 6 (Table 1). Selected spots were normalized using R package preprocessCore (Bolstad, 2018), and then differential phosphorylation was evaluated with Student’s *t*-test (*p* < 0.05). Finally, the different surfaces were compared to find the degree of similarity between them using the equation $\chi^{2}=\frac{1}{N}\,\sum_{i=1}^{N}{\left(\frac{I_{0}\,\left[i\right]-I_{t}\,\left[i\right]}{1-\left|\sigma_{0}\,\left[i\right]-\sigma_{t}\,\left[i\right]\right|}\right)}^{2}$, where χ is the degree of similarity, N is the total of spots selected, I_0_ and σ_0_ are the mean of signal intensity and standard deviation, respectively, of a group that is being compared as a model, I_t_ and σ_t_ are the mean of signal intensity and standard deviation of the test group, respectively.

**TABLE 1:** SR values for spots depending on the level of the parameters P1 and P2.

P1	P2	SR
High	High	6
High	Medium	5
Medium	High	5
High	Low	4
Low	High	4
Medium	Medium	3
Medium	Low	2
Low	Medium	2
Low	Low	1

### Bioinformatics Analysis

The connection between phosphorylated spots selected with OsteoBLAST algorithm and kinases was obtained using NetworKIN version 3.0 (Linding et al., 2007; Horn et al., 2014). The minimum score was set to 2.00, max. difference was set to 4.00 and the domains KIN, SH2, PTP and PTB were selected. Then a protein-protein interaction network (PPIN) was obtained using STRING (Szklarczyk et al., 2017) with active interaction sources as Experiments and Databases, considering minimum required interaction score as 0.400 (medium confidence). The input was proteins detected with OsteoBLAST and NetworKIN. The PPIN was analyzed using the Cytoscape (Shannon, 2003) tool NetworkAnalyzer and MCODE (Bader and Hogue, 2003) was used to screen clusters contained in the PPIN with degree cutoff = 2, node score cutoff = 0.2, k-core = 2, and max. depth = 100. Clusters with score >10 were selected for further analysis. The Database for Annotation, Visualization and Integrated Discovery (DAVID^2^) was chosen to perform Gene Ontology (Ashburner et al., 2000) and KEGG Pathways (Kanehisa et al., 2017) functional analysis.

## Results

### Surfaces Characterization

Firstly, the three different surfaces were physiochemically characterized. Supplementary Figures S4–S6 show, respectively, the secondary electron micrographs of the samples. For the Maq group, there are irregularities in the form of concentric circles related to the cutting process of the material (Supplementary Figure S5). For the DAA group (Supplementary Figure S5), the morphology is substantially altered with the appearance of craters and pores revealing removal of material. Finally, in the nanoHA group (Supplementary Figure S6) there are agglomerates that resemble the structure of corals, indicating that a coating was deposited on the surface. This structure is similar to that obtained by Bezerra et al. (2017).

The elemental composition of the surfaces, derived from EDS spectra, shows that the Maq group (Supplementary Figure S1) presents essentially Ti with low proportions of C, N, O and Fe, very possibly due to contaminations originating from the sample preparation process. Oxygen can also be due to surface oxide, spontaneously grown on metal surfaces. A very similar result is observed for the DAA group (Supplementary Figure S2), together with detection of Al (<1%). Finally, in the nanoHA group (Supplementary Figure S3), Ca and P, characteristic of hydroxyapatite, appear in addition to the already mentioned elements. The ideal Ca/P ratio in hydroxyapatite is 1.67. In the present result it was 1.71, indicating a stoichiometry very close to the HA. Supplementary Tables S1–S3 contain the concentration of elements presented in sample obtained by the analysis of EDS maps.

The geometric and harmonic surface energies of the samples are shown in Supplementary Figures S7, S8 as a function of the considered sample. Supplementary Table S4 displays the Wettablity results. Maq and DAA surfaces present practically the same surface energy value, indicating that DAA activation did not affect this property. On the other hand, the sample with nanoHA coating shows greater receptivity to other compounds and media. One interpretation for this result is proposed in terms of the presence of very receptive/reactive polar groups (OH, CO, CaCO3, CaO, etc.) in the HA structure.

### OsteoBLAST Explores Differential Kinome and Surface Similarity

**FIGURE 1:**
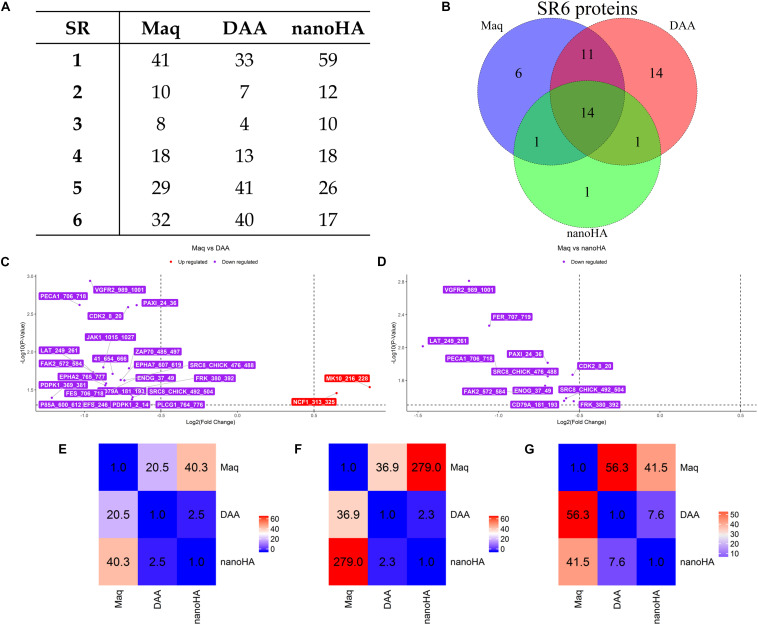
OsteoBLAST results. **(A)** SR per group summary. **(B)** Venn diagram of SR6 proteins in different groups. **(C,D)** contain the volcano-plot of DAA and nanoHA against Maq, respectively. Finally, the heat map representation of the χ parameter for pairwise comparison of all groups. The χ parameter was evaluated for SR = 6 **(E)**, SR = 4 **(F)** and SR = 2 **(G)**.

OsteoBLAST was able to determine the kinome profile of MSC during the adhesion process to different biomaterials for up to 4 h. The OsteoBLAST routine begins with an analysis of statistical parameters for the selection of reliable results. Spots were classified using the SR system, which groups them into six different levels (one is the worst and six the best). Supplementary files contain a summary of the statistical parameters of each spot for each group (Supplementary Tables S6–S8). Maq group presents 32 spots with SR 6; DAA presents 40 spots and nanoHA, 17 spots with the best SR level. Figure 1A summarizes the distribution of different SR levels in the groups. Then, spots with equal SR levels were used to perform pairwise analysis between treated and control surfaces. DAA and Maq have 25 SR 6 spots in common, while nanoHA and Maq have 15 shared SR 6 spots (Figure 1B). After the data were normalized, differential phosphorylation from the spots was evaluated using Student’s *t*-test. The DAA surface presents two spots with up-regulation of phosphorylation status and 23 with down-regulation (Figure 1C), while the nanoHA surface presents 12 spots with down-regulation of phosphorylation status (Figure 1D). The Tables 2, 3 provide additional information on the analyzed spots, such as protein, residue sequence, and phosphorylation site (p) in addition to *p*-value and fold change (FC).

**TABLE 2:** Differential phosphorylation spots of the DAA group.

Spot	Protein	Symbol	p	Uniprot accession	Sequence	*p*-value	FC
41_654_666	Protein 4.1	EPB41	[660]	P11171	LDGENIYIRHSNL	0.019426	0.568151
CD79A_181_193	B-cell antigen receptor complex-associated protein alpha chain	CD79A	[182, 188]	P11912	EYEDENLYEGLNL	0.036615	0.618438
CDK2_8_20	Cyclin-dependent kinase 2	CDK2	[15, 19]	P24941	EKIGEGTYGVVYK	0.002566	0.60936
EFS_246_258	Embryonal Fyn-associated substrate	EFS	[253]	O43281	GGTDEGIYDVPLL	0.047373	0.560947
ENOG_37_49	Gamma-enolase	ENO2	[44]	P09104	SGASTGIYEALEL	0.031402	0.565473
EPHA2_765_777	Ephrin type-A receptor 2	EPHA2	[772]	P29317	EDDPEATYTTSGG	0.027551	0.54944
EPHA7_607_619	Ephrin type-A receptor 7	EPHA7	[608, 614]	Q15375	TYIDPETYEDPNR	0.023388	0.589247
FAK2_572_584	Protein-tyrosine kinase 2-beta	PTK2B	[573, 579, 580]	Q14289	RYIEDEDYYKASV	0.025998	0.551566
FES_706_718	Tyrosine-protein kinase Fes/Fps	FES	[713]	P07332	REEADGVYAASGG	0.034676	0.561935
FRK_380_392	Tyrosine-protein kinase FRK	FRK	[387]	P42685	KVDNEDIYESRHE	0.031269	0.621248
JAK1_1015_1027	Tyrosine-protein kinase JAK1	JAK1	[1022, 1023]	P23458	AIETDKEYYTVKD	0.015949	0.544719
LAT_249_261	Linker for activation of T-cells family member 1	LAT	[255]	O43561	EEGAPDYENLQEL	0.018596	0.519961
MK10_216_228	Mitogen-activated protein kinase 10	MAPK10	[223, 228]	P53779	TSFMMTPYVVTRY	0.029102	1.819011
NCF1_313_325	Neutrophil cytosol factor 1	NCF1	[324]	P14598	QRSRKRLSQDAYR	0.034867	1.565641
P85A_600_612	Phosphatidylinositol 3-kinase regulatory subunit alpha	PIK3R1	[607]	P27986	NENTEDQYSLVED	0.045912	0.526937
PAXI_24_36	Paxillin	PXN	[31, 33]	P49023	FLSEETPYSYPTG	0.002413	0.633381
PDPK1_2_14	3-phosphoinositide-dependent protein kinase 1	PDPK1	[9]	O15530	ARTTSQLYDAVPI	0.042172	0.620032
PDPK1_369_381	3-phosphoinositide-dependent protein kinase 1	PDPK1	[373, 376]	O15530	DEDCYGNYDNLLS	0.040171	0.431449
PECA1_706_718	Platelet endothelial cell adhesion molecule	PECAM1	[713]	P16284	KKDTETVYSEVRK	0.002395	0.489083
PLCG1_764_776	1-phosphatidylinositol 4,5-bisphosphate phosphodiesterase gamma-1	PLCG1	[771, 775]	P19174	IGTAEPDYGALYE	0.048389	0.661964
SRC8_CHICK_476_488	Src substrate protein p85	CTTN1	[477, 483]	Q01406	EYEPETVYEVAGA	0.02371	0.599289
SRC8_CHICK_492_504	Src substrate protein p85	CTTN1	[492, 499, 502]	Q01406	YQAEENTYDEYEN	0.039529	0.621993
VGFR2_989_1001	Vascular endothelial growth factor receptor 2	KDR	[996]	P35968	EEAPEDLYKDFLT	0.001153	0.513102
ZAP70_485_497	Tyrosine-protein kinase ZAP-70	ZAP70	[492, 493]	P43403	ALGADDSYYTARS	0.01639	0.61186

**TABLE 3:** Differential phosphorylation spots of the nanoHA group.

Spot	Protein	Symbol	p	Uniprot accession	Sequence	*p*-value	FC
CD79A_181_193	B-cell antigen receptor complex-associated protein alpha chain	CD79A	[182, 188]	P11912	EYEDENLYEGLNL	0.0445	0.662215
CDK2_8_20	Cyclin-dependent kinase 2	CDK2	[15, 19]	P24941	EKIGEGTYGVVYK	0.02145	0.686795
ENOG_37_49	Gamma-enolase	ENO2	[44]	P09104	SGASTGIYEALEL	0.029175	0.610258
FAK2_572_584	Protein-tyrosine kinase 2-beta	PTK2B	[573, 579, 580]	Q14289	RYIEDEDYYKASV	0.03564	0.544021
FER_707_719	Tyrosine-protein kinase Fer	FER	[714]	P16591	RQEDGGVYSSSGL	0.005412	0.480022
FRK_380_392	Tyrosine-protein kinase FRK	FRK	[387]	P42685	KVDNEDIYESRHE	0.045045	0.69016
LAT_249_261	Linker for activation of T-cells family member 1	LAT	[255]	O43561	EEGAPDYENLQEL	0.009656	0.360841
PAXI_24_36	Paxillin	PXN	[31, 33]	P49023	FLSEETPYSYPTG	0.015329	0.617219
PECA1_706_718	Platelet endothelial cell adhesion molecule	PECAM1	[713]	P16284	KKDTETVYSEVRK	0.019137	0.498563
SRC8_CHICK_476_488	Src substrate protein p85	CTTN1	[477, 483]	Q01406	EYEPETVYEVAGA	0.02235	0.617239
SRC8_CHICK_492_504	Src substrate protein p85	CTTN1	[492, 499, 502]	Q01406	YQAEENTYDEYEN	0.041423	0.667946
VGFR2_989_1001	Vascular endothelial growth factor receptor 2	KDR	[996]	P35968	EEAPEDLYKDFLT	0.001543	0.440066

The comparison provides a rate of similarity between two groups, the χ parameter. For groups Maq and DAA, OsteoBLAST computed χ = 56.3 and χ = 41.5 for Maq and nanoHA groups. When comparing DAA and nanoHA, OsteoBLAST returned χ = 7.6 (Figure 1E). The χ parameter was also computed for SR = 4 and SR = 2 to exemplify the influence of the quality of spots on this comparison test. For SR = 4, Maq and DAA present χ = 36.9, Maq and nanoHA, χ = 279.0 and χ = 2.3 for DAA and nanoHA (Figure 1F). Finally, for SR = 2, Maq and DAA present χ = 20.5, Maq and nanoHA, χ = 40.3 and χ = 2.5 for DAA and nanoHA (Figure 1G).

### Network Analysis From Differential Kinome

For a better understanding of the role of differentially phosphorylated sites, a systems approach was adopted. First, the obtained sites were analyzed with NetworKIN, searching for different protein domains that interact with them. The DAA group presented 158 different proteins totaling 1641 interactions with their residues. EPHA2, EPHA7, FES, FRK, KDR, and ZAP70 were proteins that had residues with differential phosphorylation that were found as capable of interacting with other domains. The NanoHA group has 156 different proteins totaling 742 interactions with their residues. FES, FRK, and KDR were proteins that had residues with differential phosphorylation that were found as capable of interacting with other domains. Table 4 contains the interactions predicted for the spots detected with OsteoBLAST for the DAA and nanoHA groups. NetworKIN output files are in Supplementary Material.

**TABLE 4:** Interactions predicted with NetworKIN for the spots detected with OsteoBLAST.

DAA pred	Interacts with	nanoHA pred	Interacts with
EPHA2	EPHA7 (614) FRK (387) PDPK1 (373) PDPK1 (376) ZAP70 (492) LAT (255) ENO2 (44) KDR (996) CD79A (182) CD79A (188)	FES	FRK (387) LAT (255) ENO2 (44) CD79A (188)
EPHA7	EPHA7 (614) FRK (387) PDPK1 (373) PDPK1 (376) ZAP70 (492) LAT (255) ENO2 (44) KDR (996) CD79A (182) CD79A (188)	FRK	FRK (387) PTK2B (573) PTK2B (580) CDK2 (15) FER (714) LAT (255) ENO2 (44) KDR (996) CD79A (182) CD79A (188)
FES	EPHA7 (614) FRK (387) PLCG1 (775) LAT (255) ENO2 (44) CD79A (188)	KDR	FRK (387) PTK2B (580) CDK2 (19) LAT (255) KDR (996) CD79A (188)
FRK	EPHA7 (614) EFS (253) EPHA2 (772) PTK2B (573) PTK2B (580) FRK (387) PLCG1 (775) PLCG1 (771) PDPK1 (9) PDPK1 (373) PDPK1 (376) PIK3R1 (607) JAK1 (1034) JAK1 (1035) LAT (255) FES (713) ENO2 (44) CDK2 (15) KDR (996) CD79A (182) CD79A (188)		
KDR	EPHA7 (608) EPHA2 (772) PTK2B (580) FRK (387) PLCG1 (775) PLCG1 (771) PDPK1 (9) PDPK1 (373) PDPK1 (376) MAPK10 (228) PIK3R1 (607) JAK1 (1034) LAT (255) CDK2 (19) KDR (996) CD79A (188)		
ZAP70	EPHA7 (614) PLCG1 (771) ZAP70 (492) LAT (255) CD79A (182) CD79A (188)		

**FIGURE 2:**
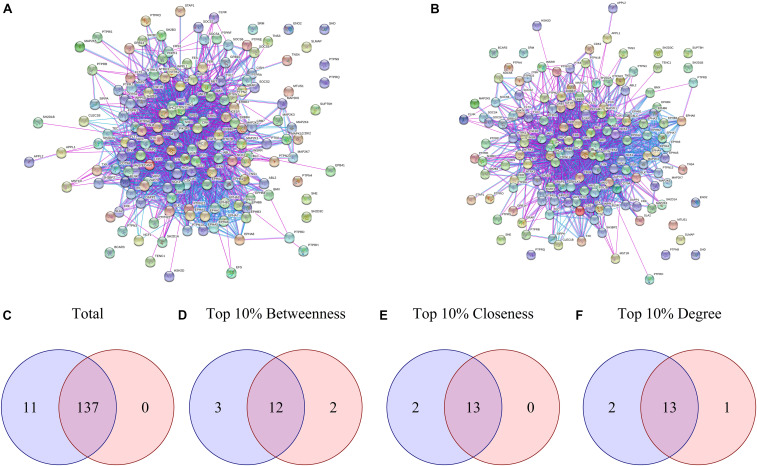
Network analysis of differential phosphorylation PPIN. **(A,B)** present the PPIN built with String for DAA and nanoHA, respectively. Venn diagrams were built to compare proteins contained in both groups based in **(C)** Total proteins, **(D)** Betweenness centrality, **(E)** Closeness centrality and **(F)** Degree of centrality. Blue represents the DAA group and red represents the nanoHA group.

Proteins presenting differential phosphorylation and their supramolecular interactors were used as input in STRING to build PPINs for each group (Figures 2A,B). The NetworkAnalyzer tool from Cytoscape analyzed both PPINs considering then as undirected networks. The DAA PPIN contains 148 proteins with 1566 interactions, while the nanoHA PPIN contains 137 proteins with 1354 interactions. NetworkAnalyzer also computed values for important network metrics as Betweenness Centrality, Closeness Centrality and Degree of Connectivity (for NetworkAnalyzer results, see Supplementary Material “*MAQDAA_node.csv*” and “*MAQNANOHA_node.csv*”). To compare the PPIN, Venn diagrams were elaborated for all proteins in the network (Figure 2C), for the upper 10th percentile of Betweenness Centrality (Figure 2D), for the upper 10th percentile of Closeness Centrality (Figure 2E) and for the upper 10th percentile of Degree of Connectivity (Figure 2F). Both PPI networks share 137 proteins, while 11 are unique for the DAA group and none are unique for nanoHA. 12 proteins are shared in the top 10% Betweenness Centrality, 3 are unique for the DAA group and 2 are unique for the nanoHA group. For top 10% Closeness Centrality, 13 proteins are shared, 2 are unique for the DAA group and none are unique for the nanoHA group. Finally, the top 10% of Degree of Connectivity presents 13 proteins in common, while 2 are unique for the DAA group and 1 is unique for the nanoHA group.

**FIGURE 3:**
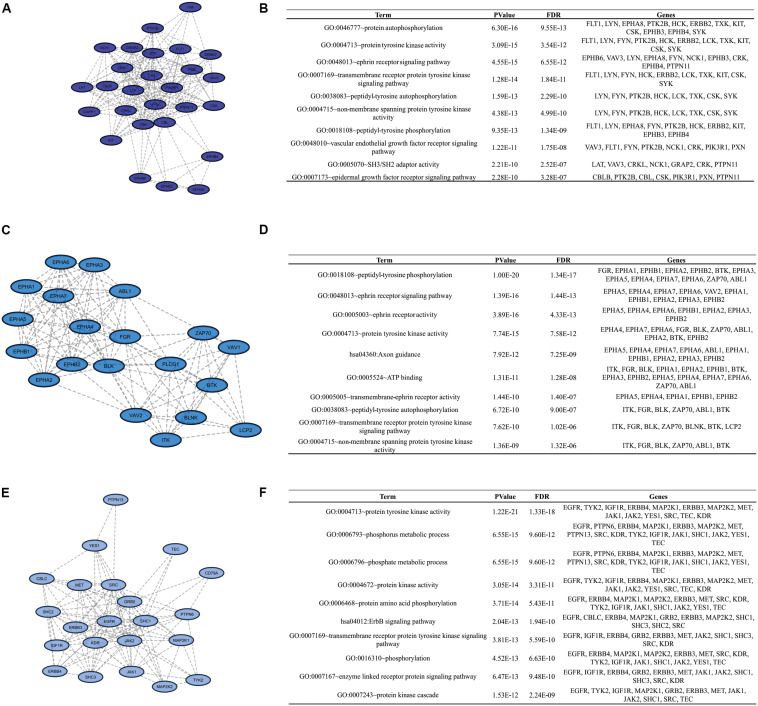
Clusters for the DAA PPIN. Letters **(A,C,E)** show the 3 clusters with MCODE score over 10. Figures **(B,D,F)** display the top DAVID IDs for each cluster.

**FIGURE 4:**
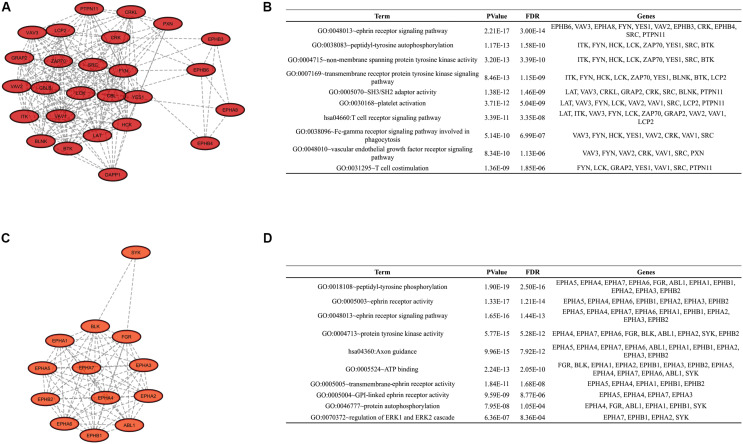
Clusters for the nanoHA PPIN. Figures **(A,C)** show the 2 clusters with MCODE score over 10. Figures **(B,D)** display the top DAVID IDs for each cluster.

To identify clusters on both PPI networks, MCODE analysis was employed. For the DAA network, 3 clusters with a score over ten were obtained. Cluster 1 with 27 proteins and score = 13.538 is composed by SYK, EPHB3, CBL, KIT, PTPN11, FYN, GRAP2, TXK, VAV3, FLT1, DAPP1, LYN, NCK1, LCK, HCK, EPHA8, PIK3R1, CSK, EPHB4, CRKL, CBLB, ERBB2, CRK, LAT, PXN, PTK2B, EPHB6 (Figure 3A). Cluster 2 has 20 proteins, score = 11.895 and is composed by PLCG1, EPHA3, ZAP70, VAV2, BLNK, BTK, EPHA5, EPHA1, LCP2, EPHA2, ABL1, EPHA6, EPHA4, ITK, FGR, VAV1, EPHB2, EPHA7, EPHB1, BLK (Figure 3C). Cluster 3 has 22 proteins, score = 10.762 and is composed by SHC3, TYK2, IGF1R, PTPN13, MAP2K2, MAP2K1, CD79A, SRC, YES1, GRB2, KDR, SHC1, EGFR, ERBB4, MET, JAK1, ERBB3, TEC, PTPN6, SHC2, CBLC, JAK2 (Figure 3E). The nanoHA network has 2 clusters with a score over 10. Cluster 1 with 26 proteins and score = 15.2 is composed by LCP2, GRAP2, CRKL, DAPP1, VAV3, CBLB, EPHA8, CRK, HCK, BTK, VAV1, PXN, BLNK, LCK, CBL, ZAP70, LAT, VAV2, SRC, ITK, EPHB3, FYN, EPHB4, YES1, EPHB6, PTPN11 (Figure 4A), while cluster 2 contains 13 proteins, score = 11.333 and is composed by EPHB2, ABL1, EPHA5, EPHA7, SYK, EPHA4, FGR, EPHA3, EPHB1, EPHA1, EPHA2, EPHA6, BLK (Figure 4C). MCODE output files are in Supplementary Material. For a better understanding of the participation of the clusters in biological processes, DAVID tools were used to perform enrichment analysis of Gene Ontology (Biological Process and Molecular Function) and KEGG Pathway analysis. The top 10 DAVID IDs are represented in Figures 3B,D,F for DAA network clusters and Figures 4B,D for nanoHA network clusters. Full DAVID results for each cluster are in Supplementary Material (“*DAVIDS_Clusters.xlsx*”).

## Discussion

With increasing life expectancy and greater urban concentration, bone lesions have been gaining greater attention in medical-dental research. In this context, biomaterials are widely used in bone regeneration, titanium being the gold standard for bone (Zambuzzi et al., 2014; Frohbergh et al., 2015; Ebrahimi et al., 2017). Many efforts have been made to modify the titanium surface, positively impacting osteoinduction and osteointegration (Nilen and Richter, 2008; Barkarmo et al., 2014; Granato et al., 2019). In this paper, we demonstrate a routine of analysis to compare different biomaterials (or any surfaces) using the kinome profile of bone marrow mesenchymal stem cells grown on three different biomaterials in order to reach the 3 Rs concept and optimize the production of biomaterials. Maq was used as a control due to its unmodified surface. DAA, a widely used surface, and nanoHA, a new product developed by SIN (Bezerra et al., 2017), were considered as test surfaces. In the last 10 years, our group has been using the analysis of intracellular signaling pathways to predict the quality of biomaterials (Zambuzzi et al., 2006, 2011a, 2014). Bezerra et al. (2017) demonstrated the biocompatibility of the surfaces using mouse pre-osteoblasts (MC3T3-E1), including its potential osteoinducer.

As a sequel to these efforts, our group presents in this work the computational routine OsteoBLAST. Composed of four steps, OsteoBLAST can provide the differential kinome between two biomaterials in order to ascribe a comparative value to each of them. In the first step, high-reliability spots were selected based on simple statistical parameters. The combination of these parameters produced a classification rate, called SR, which ranges from one to six. OsteoBLAST will only perform further analysis on groups with equal SR to avoid unbalanced data. The ideal is always to use spots with SR equal to six, which have the highest reliability, but it is possible to decrease the reliability of the analysis using spots with lower SR, if necessary. In the second step, selected spots were normalized using background correction and quantile normalization, which are frequently employed to normalize other arrays data, such as RNA-seq. Afterwards, OsteoBLAST detected spots with differential phosphorylation with fold change and *p*-value as parameters. We found 11 common spots between DAA and nanoHA: CD79A_181_193,CDK2_8_20,ENOG_37_49, FAK2_572_584, FRK_380_392, LAT_249_261, PAXI_24_36, PECA1_706_718, SRC8_CHICK_476_488, SRC8_CHICK_492_504, and VGFR2_989_1001. In a previous study, our group has demonstrated that Focal adhesion kinase 2 (FAK2) and Proto-oncogene tyrosine-protein kinase Src (SRC) are important for cell recognition of surface modifications on the nanometer scale (Zambuzzi et al., 2014). Finally, OsteoBLAST’s last step computed a χ parameter regarding the similarity between two biomaterials. If χ = 1.0, the two biomaterials have the same biological response. For the highest reliability level, χ indicates a higher similarity between the two modified surfaces (χ = 7.6) than when each was compared to the control surface (DAA χ = 56.3 and nanoHA χ = 41.5). It is also important to highlight the role of SR for a better evaluation of the χ parameter: SR = 2 and SR = 4 produced more extreme χ values, e.g., the comparison of DAA to Maq with SR = 4 resulted in χ = 279.0, while the comparison of DAA to nanoHA resulted in χ = 2.3. With more biomaterials submitted to OsteoBLAST analysis, the χ parameter range will be better defined.

In order to better understand the biological roles of the differential kinome, two questions are paramount: (1) Which residue sites are phosphorylated? (2) Which kinase phosphorylates a known phosphorylated site? PamChip and OsteoBLAST solve the first question. For the second, we choose a systemic approach. NetworKIN analysis predicts the interaction of different domains on a phosphorylated site (Linding et al., 2007; Horn et al., 2014). Some proteins used as input in NeworKIN analysis were found as predicted output (Table 4), which indicates that they promote autoregulation of the differential kinome, in some cases through autophosphorylation. A PPIN was used to define the differential kinome of a biomaterial using the sites returned by OsteoBLAST and the proteins predicted by NetworKIN. Gamma-enolase (ENO2) was the only protein with differential phosphorylation that is not part of the generated network. ENO2 is related to the calcium-dependent metabolism, catalyzes the reaction 2-phospho-D-glycerate = H2O + phosphoenolpyruvate, and its phosphorylation at Y44 has been detected by mass spectrometry (Goss et al., 2004). Platelet endothelial cell adhesion molecule (PECAM1) has shown no NetworKIN predictions for phosphorylation at the Y713 site. However, it is documented in Uniprot^3^ that the Tyrosine-protein kinase Fer phosphorylates PECAM1 at Y713 (Famiglietti et al., 1997; Paddock et al., 2011; Dasgupta et al., 2019). Protein 4.1 (EPB41) also does not present any predictions for phosphorylation at Y660 in NetworKIN results, although Uniprot reports Epidermal growth factor receptor (EGFR) as the effector kinase (Subrahmanyam et al., 1991). Some proteins predicted by NetworKIN did not present network behavior using STRING with the selected parameters.

NetworkAnalyzer returns important metrics for both PPIN. The first metric we analyzed was the Degree of Connectivity, which represents the number of interactions any single protein makes in the network. The higher the degree, the more connections the protein performs and, consequently, the greater its role in the network. Betweenness Centrality was the second metric analyzed and represents how a protein acts as an intermediate between two other proteins or, in a biological context, how much of a regulation role a protein exhibits on the network. The last metric we analyzed was Closeness Centrality, which indicates the degree each protein is close to the others. Using the top 10% quantile of these metrics as a classifier, we observe that both PPIN have more proteins in common than different ones. In fact, all 137 proteins of the nanoHA PPIN are contained in the DAA PPIN, which results in high similarity between the networks. This result corroborates the low χ value computed by OsteoBLAST for DAA and nanoHA surfaces.

Finally, enrichment analysis revealed ontologies related to phosphorylation at tyrosine residues (example: GO:0004713 ∼protein tyrosine kinase activity, GO:0038083∼peptidyl-tyrosine autophosphorylation, GO:0018108∼peptidyl-tyrosine phosphorylation, GO:0007169∼transmembrane receptor protein tyrosine kinase signaling pathway), autophosphorylation (example: GO:0046777∼protein autophosphorylation, GO:0038 083∼peptidyl-tyrosine autophosphorylation), domains SH2/SH3 (GO:0005070∼SH3/SH2 adaptor activity). These are expected ontologies since this work is based on a tyrosine kinase chip. The high amount of ontologies related to transmembrane receptors (example: GO:0007169∼transmembrane receptor protein tyrosine kinase signaling pathway, GO:0048010∼vascular endothelial growth factor receptor signaling pathway, GO:0007173∼epidermal growth factor receptor signaling pathway, GO:0048013∼ephrin receptor signaling pathway) indicates that cell metabolism during the adhesion process could be more influenced by chemical activation than physical factors, for example Integrin activation (GO:0007229∼integrin-mediated signaling pathway was also present in the results). These receptors usually undergo autophosphorylation at the beginning of the signaling cascade. Ephrin signaling-related ontologies (GO:0048013∼ephrin receptor signaling pathway, GO:0046875∼ephrin receptor binding) reveal the importance of this pathway for cell adhesion in biomaterials that has not yet been demonstrated. Ephrin type-A receptor 2 (EPHA2) and Ephrin type-A receptor 7 (EPHA7) are ephrin receptors with differential phosphorylation detected on the DAA group which were predicted by NetworKIN as potential interaction for the other sites, including the detected sites themselves, indicating autophosphorylation. Previous studies and databases confirm this information (Fang et al., 2008).

Altogether, our results demonstrate a new biomaterial analysis routine based on the differential kinome of the cell adhesion mechanism.

## Data Availability Statement

All datasets presented in this study are included in the article/Supplementary Material.

## Author Contributions

MF and WZ designed the study. WZ performed the cell cultures experiments. WZ and MP evaluated the kinase activities. MF and RM performed *in silico* simulations. MF, RM, ER, MP, and WZ analyzed the data. MF and WZ wrote the manuscript with input from all authors.

## Conflict of Interest

The authors declare that the research was conducted in the absence of any commercial or financial relationships that could be construed as a potential conflict of interest.

## Footnotes

1 https://david.ncifcrf.gov/

2 https://cran.r-project.org/

3 www.uniprot.org
