# Supplementary material for: OsteoBLAST: Computational Routine of Global Molecular Analysis Applied to Biomaterials Development
Source: Front Bioeng Biotechnol. 2020 Oct 8;8:565901. doi: 10.3389/fbioe.2020.565901 (PMC7578266; doi:10.3389/fbioe.2020.565901)
Supplement: Supplementary file 1 [file Data_Sheet_1.docx]

OsteoBLAST: computational routine of global molecular analysis applied to biomaterials development

Ferreira et al.

# Elementary composition

## Machined

Table 1 - Concentration of elements presented in sample obtained by obtained by through the analysis of EDS - Map.

| Element | Mass (%) | Atom. (%) | Sigma |
| --- | --- | --- | --- |
| Carbon (C) | 1.74 | 6.07 | 0.00 |
| Nitrogen (N) | 1.83 | 5.48 | 0.02 |
| Oxigen (O) | 2.38 | 6.23 | 0.02 |
| Titanium (Ti) | 93.66 | 81.93 | 0.27 |
| Iron (Fe) | 0.40 | 0.30 | 0.06 |


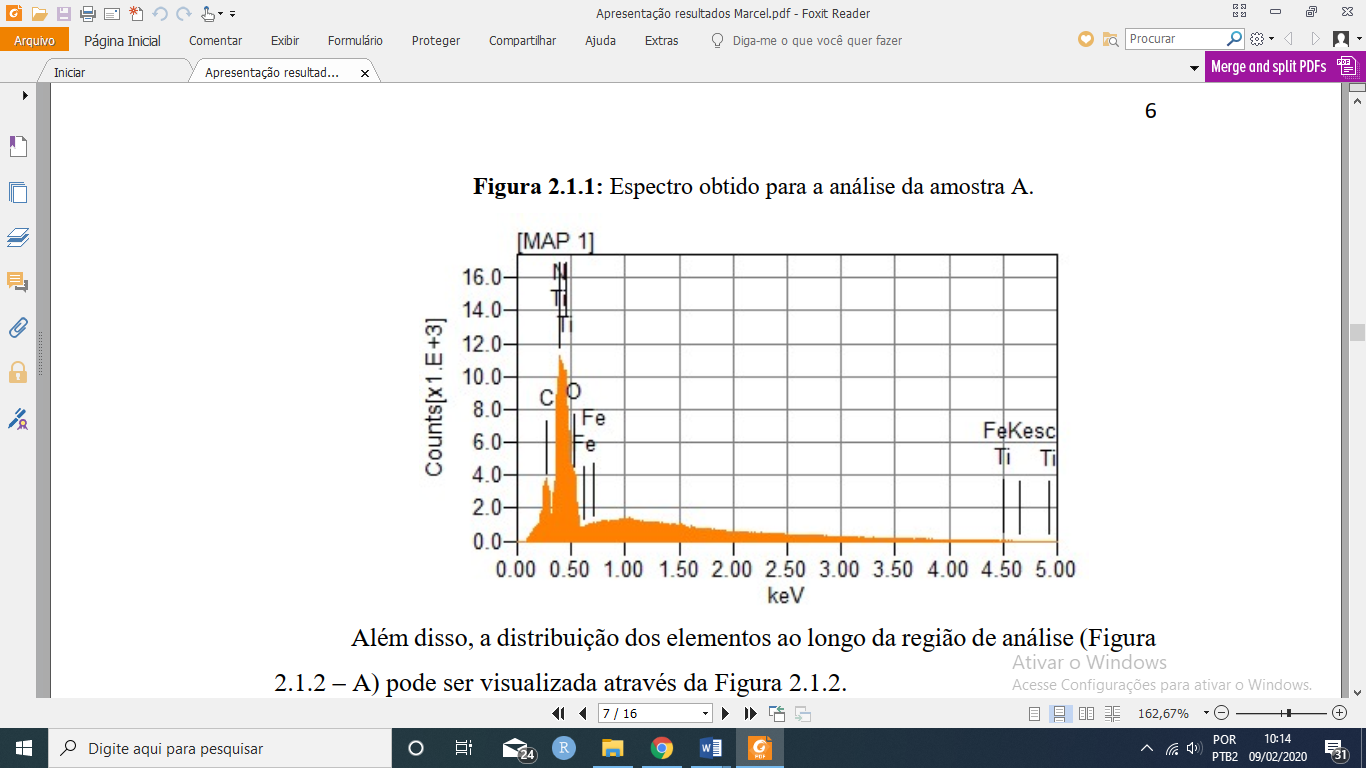


Figure 1 - Spectrum obtained from the analysis of Maq

## Dual-acid etched

Table 2 - Concentration of elements presented in sample obtained by obtained by through the analysis of EDS - Map.

| Element | Mass (%) | Atom. (%) | Sigma |
| --- | --- | --- | --- |
| Carbon (C) | 3.30 | 10.83 | 0.00 |
| Nitrogen (N) | 1.75 | 4.93 | 0.03 |
| Oxigen (O) | 3.62 | 8.91 | 0.02 |
| Aluminum (Al) | 0.62 | 0.91 | 0.02 |
| Titanium (Ti) | 89.22 | 73.37 | 0.33 |
| Iron (Fe) | 1.49 | 1.05 | 0.07 |


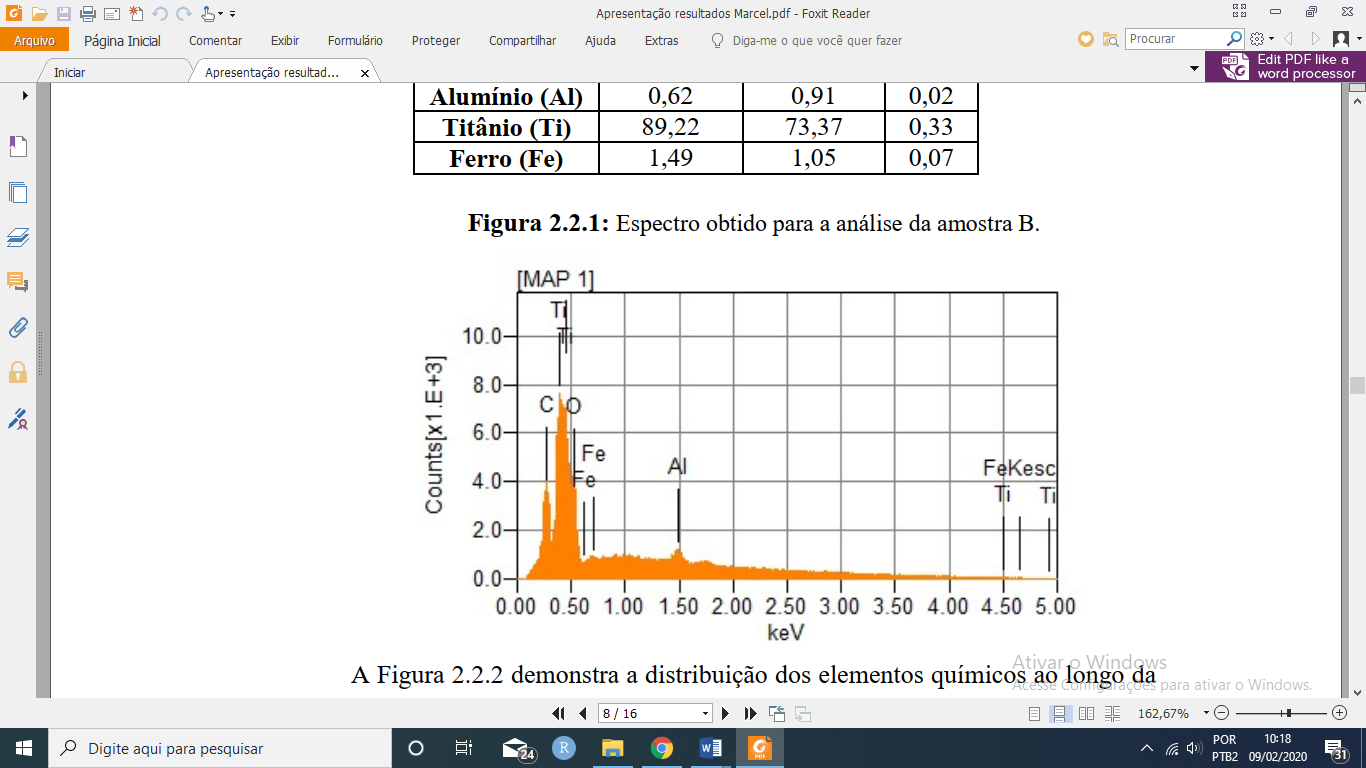


Figure 2 - Spectrum obtained from the analysis of DAA

## nanaHA

Table 3 - Concentration of elements presented in sample obtained by obtained by through the analysis of EDS - Map.

| Element | Mass (%) | Atom. (%) | Sigma |
| --- | --- | --- | --- |
| Carbon (C) | 6.11 | 15.43 | 0.01 |
| Nitrogen (N) | 4.13 | 8.96 | 0.04 |
| Oxigen (O) | 14.03 | 26.63 | 0.03 |
| Aluminum (Al) | 0.11 | 0.13 | 0.02 |
| Phosphorum (P) | 1.58 | 1.55 | 0.03 |
| Calcium (Ca) | 3.51 | 2.66 | 0.13 |
| Titanium (Ti) | 69.83 | 44.26 | 0.46 |
| Iron (Fe) | 0.68 | 0.37 | 0.08 |


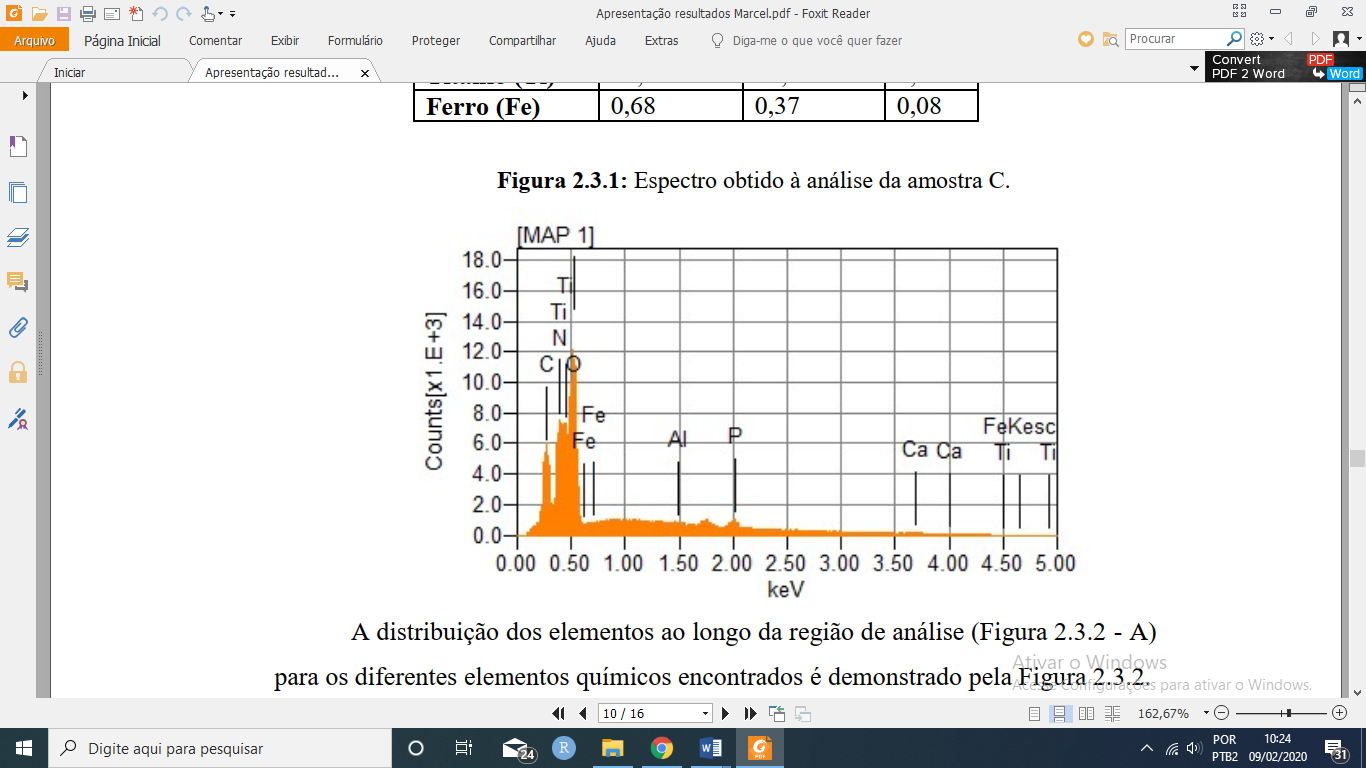


Figure 3 - Spectrum obtained from the analysis of nanoHA

# Scanning electron microscopy (SEM)


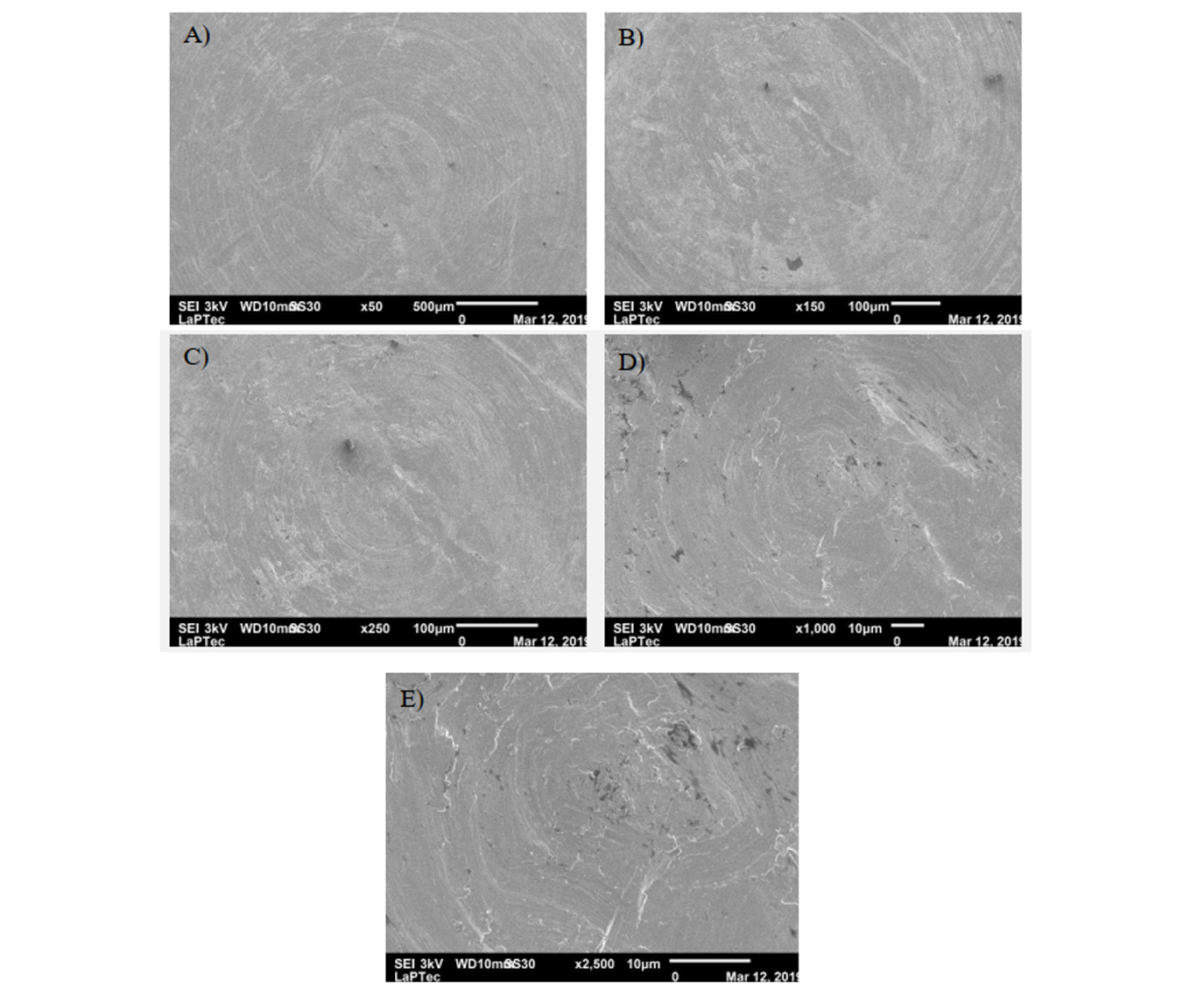


Figure 4 - Micrographs (SEM) of the machined group with magnification a) 50 X, b) 150 X, c) 250 X, d) 1000 X and e) 2500 X


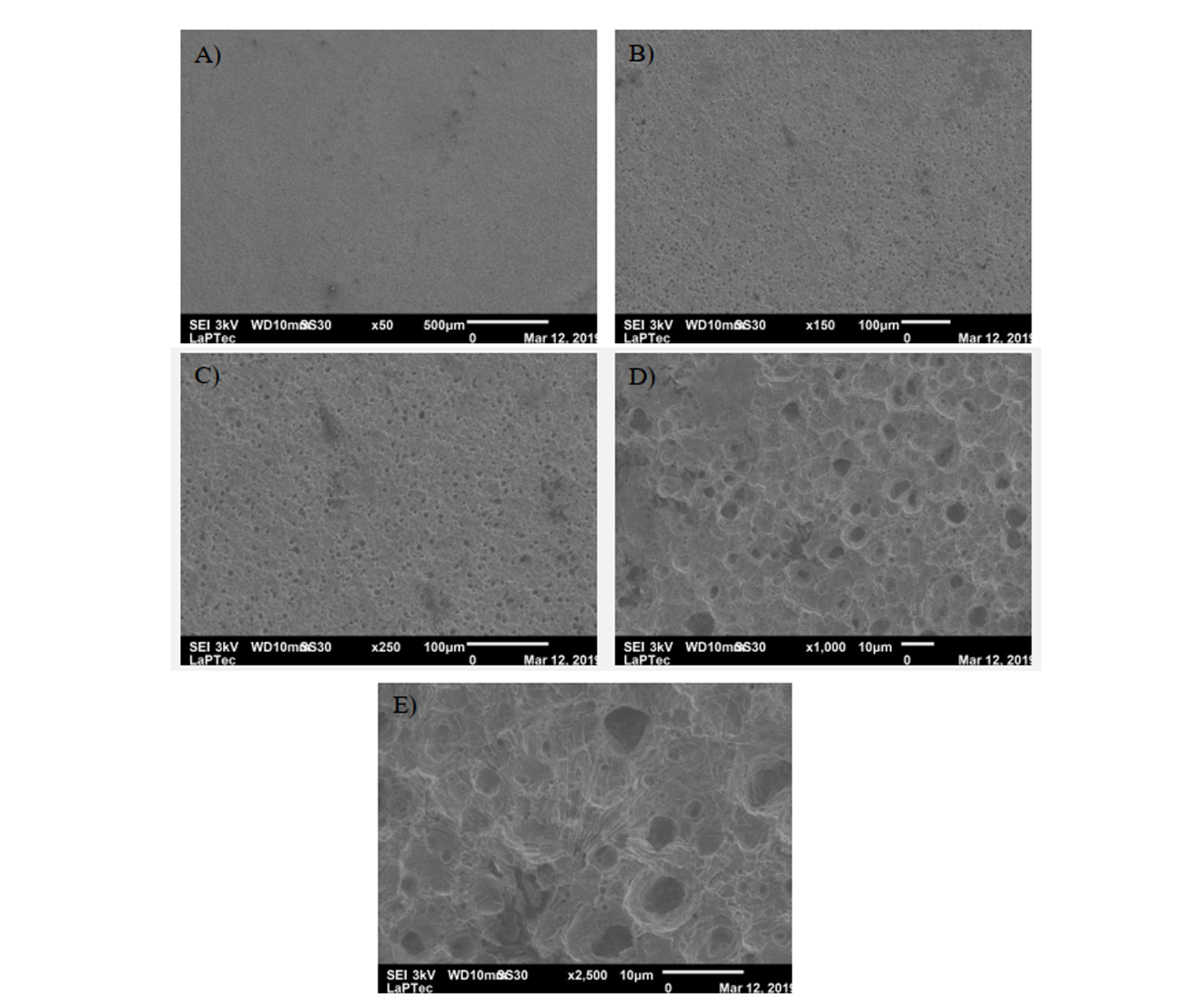


Figure 5 - Micrographs (SEM) of the dual-acid etched with magnification a) 50 X, b) 150 X, c) 250 X, d) 1000 X and e) 2500 X


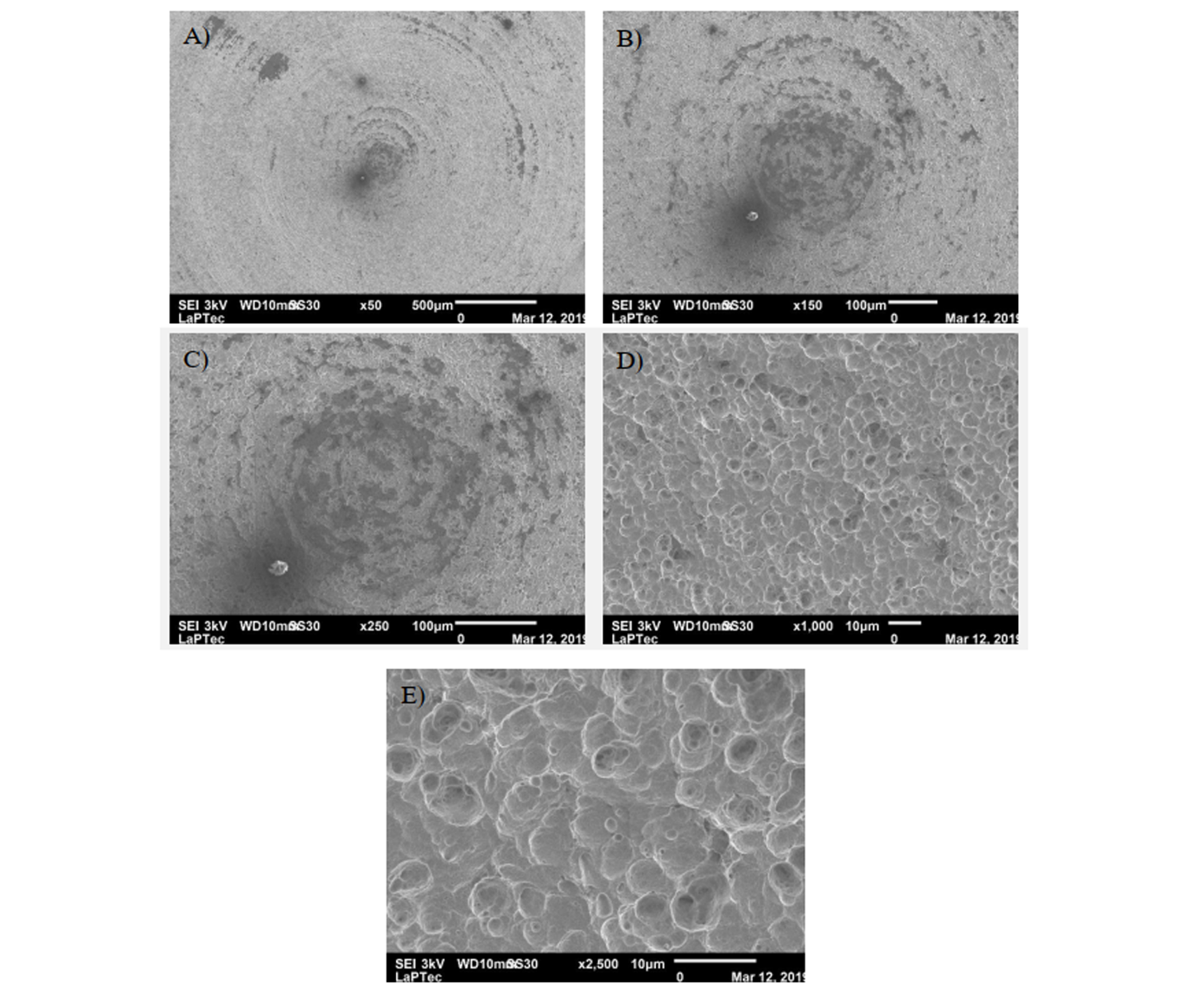


Figure 6 - Micrographs (SEM) of the nanoHA group with magnification a) 50 X, b) 150 X, c) 250 X, d) 1000 X and e) 2500 X

# Wettability

Table 4 - Mean angle and geometric and harmonic surface energy of the differents groups.

|  | Mean angle Water | Mean angle diode Methane | S.E Geometric | S.E Harmonic |
| --- | --- | --- | --- | --- |
| Machined | 81.58 ± 0.21 | 55.02 ± 0.39 | 36.18 ± 0.19 | 42.07 ± 0.18 |
| Dual-acid etched | 89.49 ± 0.86 | 44.21 ± 0.83 | 38.74 ± 0.43 | 43.11 ± 0.48 |
| nanoHA | 56.18 ± 0.36 | 46.62 ± 0.10 | 52.17 ± 0.21 | 57.51 ± 0.19 |


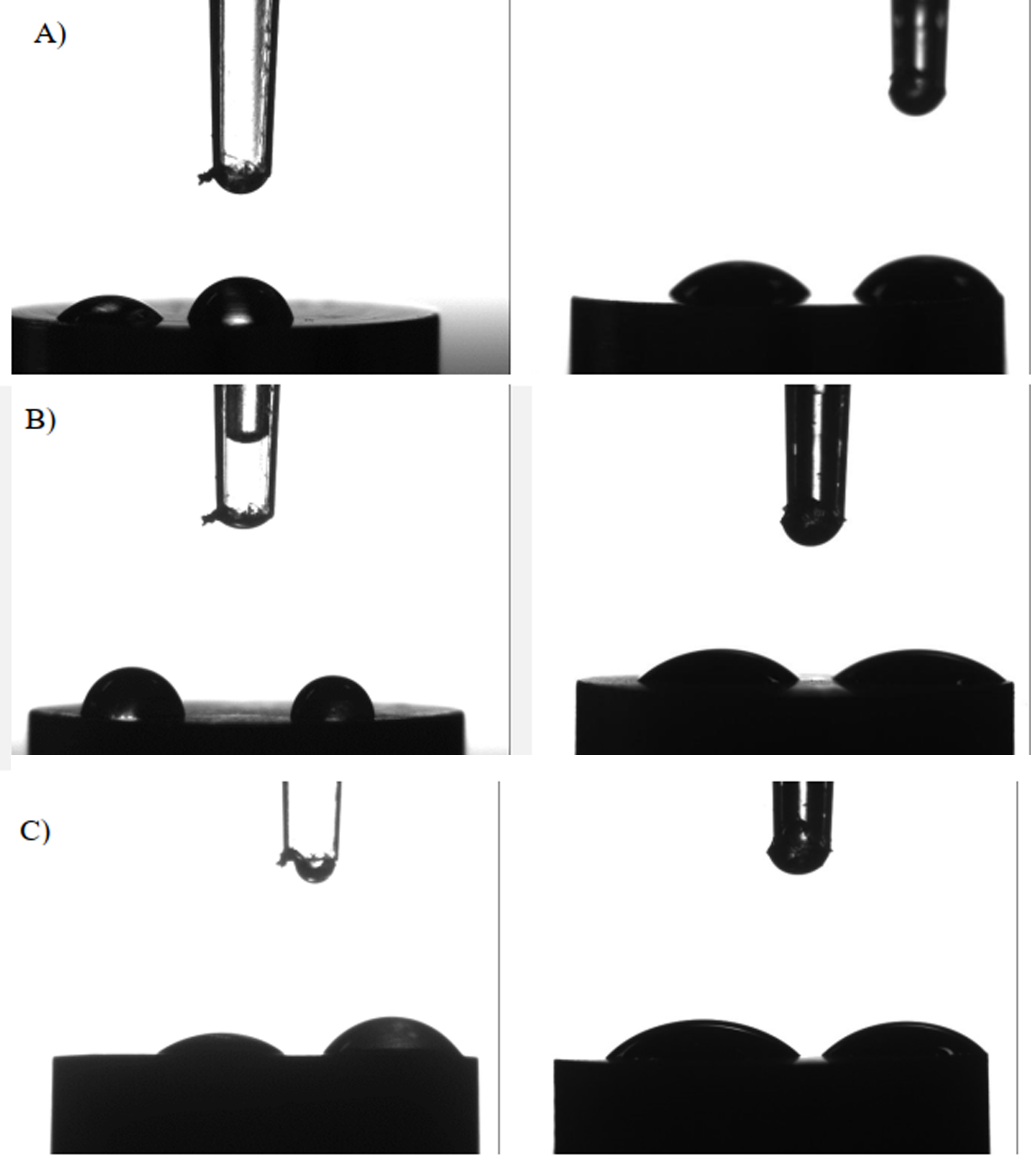


Figure 7 - Images of the drops used in the measurements of samples Machined, Dual-acid etched and nanoHA performed on the goniometer. Image of water droplets (right) and methane diode (left) A) in Machined, B) in Dual-acid etched and C) in nanoHA.


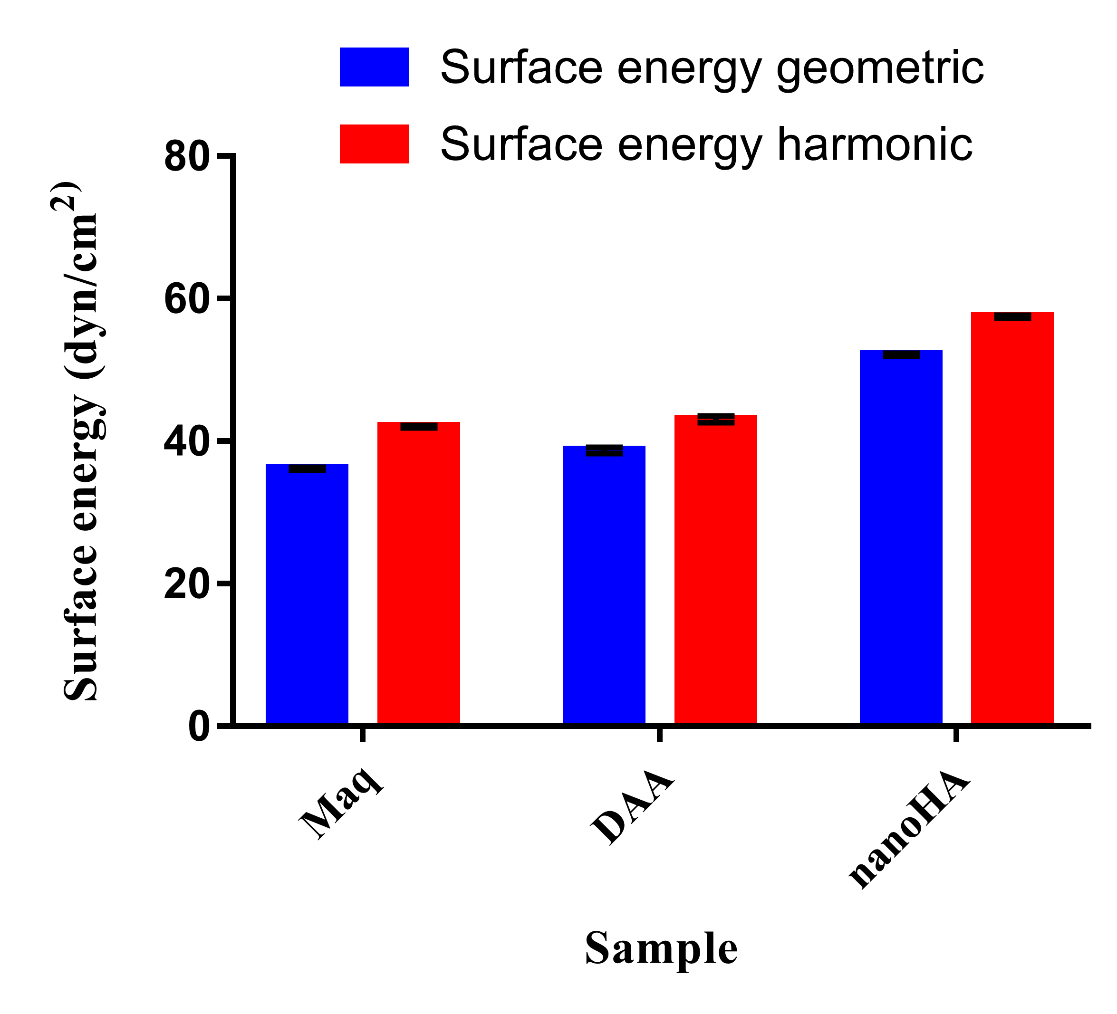


Figure 8 - Geometric and harmonic surface energy for the different groups.

# Spot relation to gene symbol (SYMBOL), phosphorylation site (P), uniprot accession (UNIPROT) and consensus phosphorylation sequence (SEQUENCE).

Table 5 –Spot features.

| spot | symbol | p | uniprot | sequence |
| --- | --- | --- | --- | --- |
| 41_654_666 | EPB41 | [660] | P11171 | LDGENIYIRHSNL |
| ACHD_383_395 | CHRND | [383, 390] | Q07001 | YISKAEEYFLLKS |
| AMPE_5_17 | ENPEP | [12] | Q07075 | EREGSKRYCIQTK |
| ANXA1_14_26 | ANXA1 | [21] | P04083 | IENEEQEYVQTVK |
| ANXA2_17_29 | ANXA2 | [24] | P07355 | HSTPPSAYGSVKA |
| ART_004_EAIYAAPFAKKKXC | ART4 | NA | NA | EAIYAAPFAKKK |
| B3AT_39_51 | SLC4A1 | [46] | P02730 | TEATATDYHTTSH |
| CTNB1_79_91 | CTNNB1 | [86] | P35222 | VADIDGQYAMTRA |
| C1R_199_211 | C1R | [204, 210] | P00736 | TEASGYISSLEYP |
| CALM_93_105 | CALM1 | [100] | P62158 | FDKDGNGYISAAE |
| PGFRB_1014_1028 | PDGFRB | [1021] | P09619 | PNEGDNDYIIPLPDP |
| ART_003_EAI(pY)AAPFAKKKXC | ART3 | NA | NA | EAI(pY)AAPFAKKK |
| CALM_95_107 | CALM1 | [100] | P62158 | KDGNGYISAAELR |
| CBL_693_705 | CBL | [700] | P22681 | EGEEDTEYMTPSS |
| CD3Z_116_128 | CD247 | [123] | P20963 | KDKMAEAYSEIGM |
| CD3Z_146_158 | CD247 | [153] | P20963 | STATKDTYDALHM |
| CD79A_181_193 | CD79A | [182, 188] | P11912 | EYEDENLYEGLNL |
| CDK2_8_20 | CDK2 | [15, 19] | P24941 | EKIGEGTYGVVYK |
| CDK7_157_169 | CDK7 | [169] | P50613 | GLAKSFGSPNRAY |
| CRK_214_226 | CRK | [221] | P46108 | GPPEPGPYAQPSV |
| DCX_109_121 | DCX | [112] | O43602 | GIVYAVSSDRFRS |
| DDR1_506_518 | DDR1 | [513] | Q08345 | LLLSNPAYRLLLA |
| DYR1A_212_224 | DYRK1A | [219, 220] | Q13627 | KHDTEMKYYIVHL |
| DYR1A_312_324 | DYRK1A | [319, 321] | Q13627 | CQLGQRIYQYIQS |
| EFS_246_258 | EFS | [253] | O43281 | GGTDEGIYDVPLL |
| EFS_246_258_Y253F | EFS | [] | O43281 | GGTDEGIFDVPLL |
| EGFR_1062_1074 | EGFR | [1069] | P00533 | EDSFLQRYSSDPT |
| EGFR_1103_1115 | EGFR | [1110] | P00533 | GSVQNPVYHNQPL |
| EGFR_1118_1130 | EGFR | [1125] | P00533 | APSRDPHYQDPHS |
| EGFR_1165_1177 | EGFR | [1172] | P00533 | ISLDNPDYQQDFF |
| EGFR_1190_1202 | EGFR | [1197] | P00533 | STAENAEYLRVAP |
| EGFR_862_874 | EGFR | [869] | P00533 | LGAEEKEYHAEGG |
| EGFR_908_920 | EGFR | [915] | P00533 | MTFGSKPYDGIPA |
| ENOG_37_49 | ENO2 | [44] | P09104 | SGASTGIYEALEL |
| EPHA1_774_786 | EPHA1 | [781] | P21709 | LDDFDGTYETQGG |
| EPHA2_581_593 | EPHA2 | [588] | P29317 | QLKPLKTYVDPHT |
| EPHA2_765_777 | EPHA2 | [772] | P29317 | EDDPEATYTTSGG |
| EPHA4_589_601 | EPHA4 | [596] | P54764 | LNQGVRTYVDPFT |
| EPHA4_921_933 | EPHA2 | [928] | P54764 | QAIKMDRYKDNFT |
| EPHA7_607_619 | EPHA7 | [608, 614] | Q15375 | TYIDPETYEDPNR |
| EPHB1_771_783 | EPHB1 | [778] | P54762 | DDTSDPTYTSSLG |
| EPHB1_921_933 | EPHB1 | [928] | P54762 | SAIKMVQYRDSFL |
| EPHB4_583_595 | EPHB4 | [590] | P54760 | IGHGTKVYIDPFT |
| EPOR_361_373 | EPOR | [368] | P19235 | SEHAQDTYLVLDK |
| EPOR_419_431 | EPOR | [426] | P19235 | ASAASFEYTILDP |
| ERBB2_1241_1253 | ERBB2 | [1248] | P04626 | PTAENPEYLGLDV |
| ERBB2_870_882 | ERBB2 | [877] | P04626 | LDIDETEYHADGG |
| ERBB4_1181_1193 | ERBB4 | [1188] | Q15303 | QALDNPEYHNASN |
| ERBB4_1277_1289 | ERBB4 | [1284] | Q15303 | IVAENPEYLSEFS |
| FABPH_13_25 | FABP3 | [20] | P05413 | DSKNFDDYMKSLG |
| FAK1_569_581 | PTK2 | [570, 576, 577] | Q05397 | RYMEDSTYYKASK |
| FAK2_572_584 | PTK2B | [573, 579, 580] | Q14289 | RYIEDEDYYKASV |
| FER_707_719 | FER | [714] | P16591 | RQEDGGVYSSSGL |
| FES_706_718 | FES | [713] | P07332 | REEADGVYAASGG |
| FGFR1_761_773 | FGFR1 | [766] | P11362 | TSNQEYLDLSMPL |
| FGFR2_762_774 | FGFR2 | [769] | P21802 | TLTTNEEYLDLSQ |
| FGFR3_641_653 | FGFR3 | [647, 648] | P22607 | DVHNLDYYKKTTN |
| FGFR3_753_765 | FGFR3 | [760] | P22607 | TVTSTDEYLDLSA |
| FRK_380_392 | FRK | [387] | P42685 | KVDNEDIYESRHE |
| INSR_1348_1360 | INSR | [1355] | P06213 | SLGFKRSYEEHIP |
| INSR_992_1004 | INSR | [992, 999] | P06213 | YASSNPEYLSASD |
| JAK1_1015_1027 | JAK1 | [1022, 1023] | P23458 | AIETDKEYYTVKD |
| JAK2_563_577 | JAK2 | [570] | O60674 | VRREVGDYGQLHETE |
| K2C6B_53_65 | KRT6B | [62] | P04259 | GAGFGSRSLYGLG |
| K2C8_425_437 | KRT8 | [427, 437] | P05787 | SAYGGLTSPGLSY |
| KSYK_518_530 | SYK | [525, 526] | P43405 | ALRADENYYKAQT |
| LAT_194_206 | LAT | [200] | O43561 | MESIDDYVNVPES |
| LAT_249_261 | LAT | [255] | O43561 | EEGAPDYENLQEL |
| LCK_387_399 | LCK | [394] | P06239 | RLIEDNEYTAREG |
| MBP_198_210 | MBP | [203] | P02686 | ARTAHYGSLPQKS |
| MBP_259_271 | MBP | [261, 268] | P02686 | FGYGGRASDYKSA |
| MBP_263_275 | MBP | [268] | P02686 | GRASDYKSAHKGF |
| MET_1227_1239 | MET | [1230, 1234, 1235] | P08581 | RDMYDKEYYSVHN |
| MK01_180_192 | MAPK1 | [187] | P28482 | HTGFLTEYVATRW |
| MK01_198_210 | MAPK1 | [205] | P28482 | IMLNSKGYTKSID |
| MK07_211_223 | MAPK7 | [215, 220] | Q13164 | AEHQYFMTEYVAT |
| MK10_216_228 | MAPK10 | [223, 228] | P53779 | TSFMMTPYVVTRY |
| MK12_178_190 | MAPK12 | [185] | P53778 | ADSEMTGYVVTRW |
| MK14_173_185 | MAPK14 | [182] | Q16539 | RHTDDEMTGYVAT |
| NCF1_313_325 | NCF1 | [324] | P14598 | QRSRKRLSQDAYR |
| NPT2A_501_513 | SLC34A1 | [511] | Q06495 | AKALGKRTAKYRW |
| NTRK1_489_501 | NTRK1 | [496] | P04629 | HIIENPQYFSDAC |
| NTRK2_509_521 | NTRK2 | [516] | Q16620 | PVIENPQYFGITN |
| NTRK2_696_708 | NTRK2 | [702, 706, 707] | Q16620 | GMSRDVYSTDYYR |
| ODBA_340_352 | BCKDHA | [345] | P12694 | DDSSAYRSVDEVN |
| ODPAT_291_303 | PDHA2 | [299] | P29803 | SMSDPGVSYRTRE |
| PP2AB_297_309 | PPP2CB | [307] | P62714 | EPHVTRRTPDYFL |
| P85A_600_612 | PIK3R1 | [607] | P27986 | NENTEDQYSLVED |
| PAXI_111_123 | PXN | [118] | P49023 | VGEEEHVYSFPNK |
| PAXI_24_36 | PXN | [31, 33] | P49023 | FLSEETPYSYPTG |
| PDPK1_2_14 | PDPK1 | [9] | O15530 | ARTTSQLYDAVPI |
| PDPK1_369_381 | PDPK1 | [373, 376] | O15530 | DEDCYGNYDNLLS |
| PECA1_706_718 | PECAM1 | [713] | P16284 | KKDTETVYSEVRK |
| PERI_458_470 | PRPH | [470] | P41219 | QRSELDKSSAHSY |
| PGFRB_1002_1014 | PDGFRB | [1009] | P09619 | LDTSSVLYTAVQP |
| PGFRB_572_584 | PDGFRB | [579, 581] | P09619 | VSSDGHEYIYVDP |
| PGFRB_709_721 | PDGFRB | [716] | P09619 | RPPSAELYSNALP |
| PGFRB_768_780 | PDGFRB | [771, 775, 778] | P09619 | SSNYMAPYDNYVP |
| PGFRB_771_783 | PDGFRB | [771, 775, 778] | P09619 | YMAPYDNYVPSAP |
| PLCG1_1246_1258 | PLCG1 | [1253] | P19174 | EGSFESRYQQPFE |
| PLCG1_764_776 | PLCG1 | [771, 775] | P19174 | IGTAEPDYGALYE |
| PLCG1_776_788 | PLCG1 | [783] | P19174 | EGRNPGFYVEANP |
| PRGR_545_557 | PGR | [557] | P06401 | LRPDSEASQSPQY |
| PRGR_786_798 | PGR | [795] | P06401 | EQRMKESSFYSLC |
| PRRX2_202_214 | PRRX2 | [208, 214] | Q99811 | WTASSPYSTVPPY |
| PTN11_539_551 | PTPN11 | [546, 551] | Q06124 | SKRKGHEYTNIKY |
| RAF1_332_344 | RAF1 | [340, 341] | P04049 | PRGQRDSSYYWEI |
| RASA1_453_465 | RASA1 | [460] | P20936 | TVDGKEIYNTIRR |
| RB_804_816 | RB1 | [805, 813] | P06400 | IYISPLKSPYKIS |
| RBL2_99_111 | RBL2 | [111] | Q08999 | VPTVSKGTVEGNY |
| RET_1022_1034 | RET | [1029] | P07949 | TPSDSLIYDDGLS |
| RET_680_692 | RET | [687] | P07949 | AQAFPVSYSSSGA |
| RON_1346_1358 | MST1R | [1353] | Q04912 | SALLGDHYVQLPA |
| RON_1353_1365 | MST1R | [1353, 1360] | Q04912 | YVQLPATYMNLGP |
| SRC8_CHICK_470_482 | CTTN1 | [477] | Q01406 | VSQREAEYEPETV |
| SRC8_CHICK_476_488 | CTTN1 | [477, 483] | Q01406 | EYEPETVYEVAGA |
| SRC8_CHICK_492_504 | CTTN1 | [492, 499, 502] | Q01406 | YQAEENTYDEYEN |
| STA5A_687_699 | STAT5A | [694] | P42229 | LAKAVDGYVKPQI |
| STAT1_694_706 | STAT1 | [701] | P42224 | DGPKGTGYIKTEL |
| STAT3_698_710 | STAT3 | [705] | P40763 | DPGSAAPYLKTKF |
| STAT4_686_698 | STAT4 | [693] | Q14765 | TERGDKGYVPSVF |
| STAT4_714_726 | STAT4 | [725] | Q14765 | PSDLLPMSPSVYA |
| STAT6_634_646 | STAT6 | [641] | P42226 | MGKDGRGYVPATI |
| TEC_512_524 | TEC | [513, 519] | P42680 | RYFLDDQYTSSSG |
| TNNT1_2_14 | TNNT1 | [9] | P13805 | SDTEEQEYEEEQP |
| TYRO3_679_691 | TYRO3 | [681, 685, 686] | Q06418 | KIYSGDYYRQGCA |
| VGFR1_1040_1052 | FLT1 | [1048] | P17948 | DFGLARDIYKNPD |
| VGFR1_1046_1058_Y1048F | FLT1 | [1053] | P17948 | DIFKNPDYVRKGD |
| VGFR1_1049_1061 | FLT1 | [1053] | P17948 | KNPDYVRKGDTRL |
| VGFR1_1162_1174 | FLT1 | [1169] | P17948 | VQQDGKDYIPINA |
| VGFR1_1206_1218 | FLT1 | [1213] | P17948 | GSSDDVRYVNAFK |
| VGFR1_1235_1247 | FLT1 | [1242] | P17948 | ATSMFDDYQGDSS |
| VGFR1_1320_1332_C1320S/C1321S | FLT1 | [1327] | P17948 | SSSPPPDYNSVVL |
| VGFR1_1326_1338 | FLT1 | [1327, 1333] | P17948 | DYNSVVLYSTPPI |
| VGFR2_1046_1058 | KDR | [1054] | P35968 | DFGLARDIYKDPD |
| VGFR2_1052_1064 | KDR | [1054, 1059] | P35968 | DIYKDPDYVRKGD |
| VGFR2_1168_1180 | KDR | [1175] | P35968 | AQQDGKDYIVLPI |
| VGFR2_1207_1219_C1208S | KDR | [1214] | P35968 | VSDPKFHYDNTAG |
| VGFR2_944_956 | KDR | [951] | P35968 | RFRQGKDYVGAIP |
| VGFR2_989_1001 | KDR | [996] | P35968 | EEAPEDLYKDFLT |
| VGFR3_1061_1073 | FLT4 | [1063, 1068] | P35916 | DIYKDPDYVRKGS |
| VINC_815_827 | VCL | [822] | P18206 | KSFLDSGYRILGA |
| ZAP70_485_497 | ZAP70 | [492, 493] | P43403 | ALGADDSYYTARS |
| ZBT16_621_633 | ZBTB16 | [630] | Q05516 | LRTHNGASPYQCT |

# Stats from Maq group

Table 6 - Summary of the statistical parameters of each spot for Maq group.

| spot | A | M | SD | P1 | P2 |
| --- | --- | --- | --- | --- | --- |
| 41_654_666 | 83.33333 | 89 | 10.69268 | 0.128312 | 0.93633 |
| ACHD_383_395 | 4.333333 | 1 | 6.658328 | 1.536537 | 4.333333 |
| AMPE_5_17 | 7.333333 | 10 | 4.618802 | 0.629837 | 0.733333 |
| ANXA1_14_26 | 36.66667 | 30 | 14.22439 | 0.387938 | 1.222222 |
| ANXA2_17_29 | 5.333333 | 5 | 5.507571 | 1.032669 | 1.066667 |
| ART_004_EAIYAAPFAKKKXC | 473.3333 | 447 | 123.6217 | 0.261173 | 1.058911 |
| B3AT_39_51 | 1.333333 | 0 | 2.309401 | 1.732051 | Inf |
| CTNB1_79_91 | 16.33333 | 18 | 9.609024 | 0.588308 | 0.907407 |
| C1R_199_211 | 4.333333 | 2 | 4.041452 | 0.932643 | 2.166667 |
| CALM_93_105 | 1 | 0 | 1.732051 | 1.732051 | Inf |
| PGFRB_1014_1028 | 42.33333 | 40 | 23.58672 | 0.557167 | 1.058333 |
| ART_003_EAI(pY)AAPFAKKKXC | 1356 | 1305 | 558.2499 | 0.411689 | 1.03908 |
| CALM_95_107 | 4.333333 | 4 | 2.516611 | 0.580756 | 1.083333 |
| CBL_693_705 | 23.66667 | 21 | 5.507571 | 0.232714 | 1.126984 |
| CD3Z_116_128 | 6 | 0 | 10.3923 | 1.732051 | Inf |
| CD3Z_146_158 | 0.666667 | 0 | 1.154701 | 1.732051 | Inf |
| CD79A_181_193 | 1907.667 | 1876 | 296.7698 | 0.155567 | 1.01688 |
| CDK2_8_20 | 184 | 183 | 20.51828 | 0.111512 | 1.005464 |
| CDK7_157_169 | 28.66667 | 27 | 10.59874 | 0.369724 | 1.061728 |
| CRK_214_226 | 15.66667 | 17 | 8.082904 | 0.51593 | 0.921569 |
| DCX_109_121 | 54.66667 | 46 | 20.42874 | 0.373696 | 1.188406 |
| DDR1_506_518 | 5.666667 | 0 | 9.814955 | 1.732051 | Inf |
| DYR1A_212_224 | 0.666667 | 0 | 1.154701 | 1.732051 | Inf |
| DYR1A_312_324 | 21.33333 | 16 | 15.69501 | 0.735704 | 1.333333 |
| EFS_246_258 | 704.3333 | 723 | 134.9716 | 0.19163 | 0.974182 |
| EFS_246_258_Y253F | 0.333333 | 0 | 0.57735 | 1.732051 | Inf |
| EGFR_1062_1074 | 6 | 4 | 6.244998 | 1.040833 | 1.5 |
| EGFR_1103_1115 | 13 | 13 | 7 | 0.538462 | 1 |
| EGFR_1118_1130 | 6.333333 | 5 | 4.163332 | 0.657368 | 1.266667 |
| EGFR_1165_1177 | 39.33333 | 40 | 5.033223 | 0.127963 | 0.983333 |
| EGFR_1190_1202 | 1.666667 | 0 | 2.886751 | 1.732051 | Inf |
| EGFR_862_874 | 0 | 0 | 0 | NA | NA |
| EGFR_908_920 | 1 | 0 | 1.732051 | 1.732051 | Inf |
| ENOG_37_49 | 701.3333 | 684 | 108.0478 | 0.154061 | 1.025341 |
| EPHA1_774_786 | 154.3333 | 136 | 37.072 | 0.240207 | 1.134804 |
| EPHA2_581_593 | 2.333333 | 0 | 4.041452 | 1.732051 | Inf |
| EPHA2_765_777 | 165.6667 | 153 | 31.94266 | 0.192813 | 1.082789 |
| EPHA4_589_601 | 18.66667 | 16 | 6.429101 | 0.344416 | 1.166667 |
| EPHA4_921_933 | 3.666667 | 2 | 4.725816 | 1.288859 | 1.833333 |
| EPHA7_607_619 | 80.66667 | 77 | 11.93035 | 0.147897 | 1.047619 |
| EPHB1_771_783 | 58.33333 | 48 | 19.65536 | 0.336949 | 1.215278 |
| EPHB1_921_933 | 37.33333 | 28 | 16.16581 | 0.433013 | 1.333333 |
| EPHB4_583_595 | 7.333333 | 10 | 6.429101 | 0.876696 | 0.733333 |
| EPOR_361_373 | 41 | 33 | 14.73092 | 0.359291 | 1.242424 |
| EPOR_419_431 | 41.66667 | 34 | 16.86219 | 0.404692 | 1.22549 |
| ERBB2_1241_1253 | 25.66667 | 23 | 13.20353 | 0.514423 | 1.115942 |
| ERBB2_870_882 | 25.33333 | 20 | 13.79613 | 0.544584 | 1.266667 |
| ERBB4_1181_1193 | 3 | 0 | 5.196152 | 1.732051 | Inf |
| ERBB4_1277_1289 | 31.66667 | 33 | 6.110101 | 0.192951 | 0.959596 |
| FABPH_13_25 | 0 | 0 | 0 | NA | NA |
| FAK1_569_581 | 21.33333 | 21 | 2.516611 | 0.117966 | 1.015873 |
| FAK2_572_584 | 55.66667 | 58 | 4.932883 | 0.088615 | 0.95977 |
| FER_707_719 | 66.66667 | 67 | 9.504385 | 0.142566 | 0.995025 |
| FES_706_718 | 110 | 99 | 20.80865 | 0.18917 | 1.111111 |
| FGFR1_761_773 | 6.666667 | 8 | 6.110101 | 0.916515 | 0.833333 |
| FGFR2_762_774 | 32.66667 | 35 | 8.736895 | 0.267456 | 0.933333 |
| FGFR3_641_653 | 0.333333 | 0 | 0.57735 | 1.732051 | Inf |
| FGFR3_753_765 | 24.33333 | 24 | 11.50362 | 0.472752 | 1.013889 |
| FRK_380_392 | 367.3333 | 355 | 51.61718 | 0.140519 | 1.034742 |
| INSR_1348_1360 | 8 | 7 | 8.544004 | 1.068 | 1.142857 |
| INSR_992_1004 | 13.66667 | 12 | 6.658328 | 0.487195 | 1.138889 |
| JAK1_1015_1027 | 31.33333 | 32 | 5.033223 | 0.160635 | 0.979167 |
| JAK2_563_577 | 67 | 60 | 19.46792 | 0.290566 | 1.116667 |
| K2C6B_53_65 | 48 | 51 | 14.73092 | 0.306894 | 0.941176 |
| K2C8_425_437 | 7.333333 | 5 | 7.767453 | 1.059198 | 1.466667 |
| KSYK_518_530 | 12.33333 | 14 | 7.637626 | 0.619267 | 0.880952 |
| LAT_194_206 | 18.33333 | 16 | 16.62328 | 0.906724 | 1.145833 |
| LAT_249_261 | 83.33333 | 82 | 7.094599 | 0.085135 | 1.01626 |
| LCK_387_399 | 33 | 28 | 14.17745 | 0.42962 | 1.178571 |
| MBP_198_210 | 32 | 30 | 6.244998 | 0.195156 | 1.066667 |
| MBP_259_271 | 8.333333 | 7 | 5.131601 | 0.615792 | 1.190476 |
| MBP_263_275 | 6.333333 | 8 | 5.686241 | 0.897827 | 0.791667 |
| MET_1227_1239 | 45.33333 | 47 | 3.785939 | 0.083513 | 0.964539 |
| MK01_180_192 | 17.66667 | 14 | 10.01665 | 0.56698 | 1.261905 |
| MK01_198_210 | 2.666667 | 0 | 4.618802 | 1.732051 | Inf |
| MK07_211_223 | 4 | 0 | 6.928203 | 1.732051 | Inf |
| MK10_216_228 | 47 | 43 | 11.53256 | 0.245374 | 1.093023 |
| MK12_178_190 | 8 | 5 | 5.196152 | 0.649519 | 1.6 |
| MK14_173_185 | 2.666667 | 0 | 4.618802 | 1.732051 | Inf |
| NCF1_313_325 | 54 | 51 | 7 | 0.12963 | 1.058824 |
| NPT2A_501_513 | 7.333333 | 3 | 10.21437 | 1.392868 | 2.444444 |
| NTRK1_489_501 | 7.333333 | 6 | 8.082904 | 1.102214 | 1.222222 |
| NTRK2_509_521 | 0 | 0 | 0 | NA | NA |
| NTRK2_696_708 | 50 | 52 | 9.165151 | 0.183303 | 0.961538 |
| ODBA_340_352 | 14.66667 | 10 | 11.71893 | 0.799018 | 1.466667 |
| ODPAT_291_303 | 1.666667 | 1 | 2.081666 | 1.249 | 1.666667 |
| PP2AB_297_309 | 35.66667 | 39 | 8.504901 | 0.238455 | 0.91453 |
| P85A_600_612 | 182 | 163 | 40.03748 | 0.219986 | 1.116564 |
| PAXI_111_123 | 235.6667 | 215 | 39.31073 | 0.166806 | 1.096124 |
| PAXI_24_36 | 164.6667 | 169 | 9.291573 | 0.056427 | 0.974359 |
| PDPK1_2_14 | 78.33333 | 71 | 15.37314 | 0.196253 | 1.103286 |
| PDPK1_369_381 | 72.66667 | 63 | 18.50225 | 0.254618 | 1.153439 |
| PECA1_706_718 | 113.6667 | 119 | 10.11599 | 0.088997 | 0.955182 |
| PERI_458_470 | 1 | 0 | 1.732051 | 1.732051 | Inf |
| PGFRB_1002_1014 | 30.66667 | 26 | 18.44813 | 0.601569 | 1.179487 |
| PGFRB_572_584 | 179 | 164 | 35.93049 | 0.200729 | 1.091463 |
| PGFRB_709_721 | 34.33333 | 34 | 10.50397 | 0.305941 | 1.009804 |
| PGFRB_768_780 | 19.66667 | 15 | 8.082904 | 0.410995 | 1.311111 |
| PGFRB_771_783 | 34.66667 | 29 | 10.69268 | 0.308443 | 1.195402 |
| PLCG1_1246_1258 | 7.666667 | 5 | 4.618802 | 0.602452 | 1.533333 |
| PLCG1_764_776 | 385.6667 | 359 | 67.09943 | 0.173983 | 1.07428 |
| PLCG1_776_788 | 1.333333 | 0 | 2.309401 | 1.732051 | Inf |
| PRGR_545_557 | 1.333333 | 0 | 2.309401 | 1.732051 | Inf |
| PRGR_786_798 | 3.333333 | 0 | 5.773503 | 1.732051 | Inf |
| PRRX2_202_214 | 31 | 33 | 4.358899 | 0.14061 | 0.939394 |
| PTN11_539_551 | 15.66667 | 13 | 6.429101 | 0.410368 | 1.205128 |
| RAF1_332_344 | 92.33333 | 91 | 17.03917 | 0.18454 | 1.014652 |
| RASA1_453_465 | 45.66667 | 43 | 5.507571 | 0.120604 | 1.062016 |
| RB_804_816 | 27.66667 | 27 | 12.01388 | 0.434237 | 1.024691 |
| RBL2_99_111 | 1.333333 | 0 | 2.309401 | 1.732051 | Inf |
| RET_1022_1034 | 151 | 147 | 31.19295 | 0.206576 | 1.027211 |
| RET_680_692 | 0.666667 | 0 | 1.154701 | 1.732051 | Inf |
| RON_1346_1358 | 22 | 19 | 9.848858 | 0.447675 | 1.157895 |
| RON_1353_1365 | 11.33333 | 13 | 9.609024 | 0.847855 | 0.871795 |
| SRC8_CHICK_470_482 | 2.666667 | 2 | 3.05505 | 1.145644 | 1.333333 |
| SRC8_CHICK_476_488 | 605.3333 | 565 | 80.47567 | 0.132944 | 1.071386 |
| SRC8_CHICK_492_504 | 768.3333 | 726 | 113.5796 | 0.147826 | 1.05831 |
| STA5A_687_699 | 1.333333 | 0 | 2.309401 | 1.732051 | Inf |
| STAT1_694_706 | 0 | 0 | 0 | NA | NA |
| STAT3_698_710 | 4.666667 | 0 | 8.082904 | 1.732051 | Inf |
| STAT4_686_698 | 5.666667 | 4 | 6.658328 | 1.174999 | 1.416667 |
| STAT4_714_726 | 6.333333 | 0 | 10.96966 | 1.732051 | Inf |
| STAT6_634_646 | 1.666667 | 0 | 2.886751 | 1.732051 | Inf |
| TEC_512_524 | 28.66667 | 26 | 9.291573 | 0.324125 | 1.102564 |
| TNNT1_2_14 | 4.666667 | 0 | 8.082904 | 1.732051 | Inf |
| TYRO3_679_691 | 39 | 38 | 2.645751 | 0.06784 | 1.026316 |
| VGFR1_1040_1052 | 10 | 6 | 10.58301 | 1.058301 | 1.666667 |
| VGFR1_1046_1058_Y1048F | 1.666667 | 2 | 1.527525 | 0.916515 | 0.833333 |
| VGFR1_1049_1061 | 4 | 5 | 3.605551 | 0.901388 | 0.8 |
| VGFR1_1162_1174 | 0 | 0 | 0 | NA | NA |
| VGFR1_1206_1218 | 1 | 0 | 1.732051 | 1.732051 | Inf |
| VGFR1_1235_1247 | 0 | 0 | 0 | NA | NA |
| VGFR1_1320_1332_C1320S/C1321S | 4 | 1 | 5.196152 | 1.299038 | 4 |
| VGFR1_1326_1338 | 28.66667 | 28 | 10.01665 | 0.349418 | 1.02381 |
| VGFR2_1046_1058 | 23.66667 | 18 | 10.69268 | 0.451803 | 1.314815 |
| VGFR2_1052_1064 | 20 | 20 | 8 | 0.4 | 1 |
| VGFR2_1168_1180 | 10.33333 | 12 | 9.609024 | 0.929906 | 0.861111 |
| VGFR2_1207_1219_C1208S | 1 | 0 | 1.732051 | 1.732051 | Inf |
| VGFR2_944_956 | 38.33333 | 36 | 11.67619 | 0.304596 | 1.064815 |
| VGFR2_989_1001 | 122.3333 | 117 | 10.11599 | 0.082692 | 1.045584 |
| VGFR3_1061_1073 | 5 | 5 | 5 | 1 | 1 |
| VINC_815_827 | 15 | 16 | 11.53256 | 0.768838 | 0.9375 |
| ZAP70_485_497 | 35 | 35 | 9 | 0.257143 | 1 |
| ZBT16_621_633 | 10.66667 | 11 | 2.516611 | 0.235932 | 0.969697 |

# Stats from DAA group

Table 7 - Summary of the statistical parameters of each spot for DAA group.

| spot | A | M | SD | P1 | P2 |
| --- | --- | --- | --- | --- | --- |
| 41_654_666 | 57.66667 | 57 | 3.05505 | 0.052978 | 1.011696 |
| ACHD_383_395 | 5 | 3 | 6.244998 | 1.249 | 1.666667 |
| AMPE_5_17 | 3.666667 | 3 | 4.041452 | 1.102214 | 1.222222 |
| ANXA1_14_26 | 36.66667 | 34 | 6.429101 | 0.175339 | 1.078431 |
| ANXA2_17_29 | 10.33333 | 10 | 1.527525 | 0.147825 | 1.033333 |
| ART_004_EAIYAAPFAKKKXC | 366.6667 | 338 | 89.51164 | 0.244123 | 1.084813 |
| B3AT_39_51 | 2 | 2 | 2 | 1 | 1 |
| CTNB1_79_91 | 13.66667 | 14 | 7.505553 | 0.549187 | 0.97619 |
| C1R_199_211 | 11 | 7 | 6.928203 | 0.629837 | 1.571429 |
| CALM_93_105 | 3.666667 | 3 | 4.041452 | 1.102214 | 1.222222 |
| PGFRB_1014_1028 | 35 | 37 | 13.11488 | 0.374711 | 0.945946 |
| ART_003_EAI(pY)AAPFAKKKXC | 1342 | 1290 | 357.8449 | 0.26665 | 1.04031 |
| CALM_95_107 | 8.666667 | 6 | 4.618802 | 0.532939 | 1.444444 |
| CBL_693_705 | 16.33333 | 13 | 14.29452 | 0.875175 | 1.25641 |
| CD3Z_116_128 | 3.333333 | 0 | 5.773503 | 1.732051 | Inf |
| CD3Z_146_158 | 1 | 0 | 1.732051 | 1.732051 | Inf |
| CD79A_181_193 | 1188.667 | 1197 | 112.7312 | 0.094838 | 0.993038 |
| CDK2_8_20 | 121.3333 | 124 | 15.17674 | 0.125083 | 0.978495 |
| CDK7_157_169 | 33.66667 | 37 | 7.571878 | 0.224907 | 0.90991 |
| CRK_214_226 | 15.66667 | 18 | 4.932883 | 0.314865 | 0.87037 |
| DCX_109_121 | 60 | 60 | 3 | 0.05 | 1 |
| DDR1_506_518 | 1.333333 | 0 | 2.309401 | 1.732051 | Inf |
| DYR1A_212_224 | 0 | 0 | 0 | NA | NA |
| DYR1A_312_324 | 15.33333 | 14 | 6.110101 | 0.398485 | 1.095238 |
| EFS_246_258 | 406 | 404 | 37.04052 | 0.091233 | 1.00495 |
| EFS_246_258_Y253F | 1 | 0 | 1.732051 | 1.732051 | Inf |
| EGFR_1062_1074 | 8 | 5 | 9.848858 | 1.231107 | 1.6 |
| EGFR_1103_1115 | 14.33333 | 17 | 7.371115 | 0.514264 | 0.843137 |
| EGFR_1118_1130 | 3.333333 | 3 | 1.527525 | 0.458258 | 1.111111 |
| EGFR_1165_1177 | 43.66667 | 48 | 7.505553 | 0.171883 | 0.909722 |
| EGFR_1190_1202 | 0 | 0 | 0 | NA | NA |
| EGFR_862_874 | 1.333333 | 0 | 2.309401 | 1.732051 | Inf |
| EGFR_908_920 | 1 | 0 | 1.732051 | 1.732051 | Inf |
| ENOG_37_49 | 407.3333 | 404 | 23.18045 | 0.056908 | 1.008251 |
| EPHA1_774_786 | 108.6667 | 113 | 10.21437 | 0.093997 | 0.961652 |
| EPHA2_581_593 | 2 | 0 | 3.464102 | 1.732051 | Inf |
| EPHA2_765_777 | 102.3333 | 95 | 14.46836 | 0.141385 | 1.077193 |
| EPHA4_589_601 | 26 | 29 | 5.196152 | 0.199852 | 0.896552 |
| EPHA4_921_933 | 8 | 5 | 7 | 0.875 | 1.6 |
| EPHA7_607_619 | 57 | 52 | 12.28821 | 0.215583 | 1.096154 |
| EPHB1_771_783 | 41 | 36 | 11.35782 | 0.27702 | 1.138889 |
| EPHB1_921_933 | 53.33333 | 50 | 12.34234 | 0.231419 | 1.066667 |
| EPHB4_583_595 | 9.333333 | 11 | 3.785939 | 0.405636 | 0.848485 |
| EPOR_361_373 | 25.33333 | 23 | 8.736895 | 0.344877 | 1.101449 |
| EPOR_419_431 | 32.33333 | 28 | 8.386497 | 0.259376 | 1.154762 |
| ERBB2_1241_1253 | 26 | 25 | 8.544004 | 0.328616 | 1.04 |
| ERBB2_870_882 | 25.66667 | 31 | 11.01514 | 0.429161 | 0.827957 |
| ERBB4_1181_1193 | 4 | 2 | 5.291503 | 1.322876 | 2 |
| ERBB4_1277_1289 | 30 | 25 | 9.539392 | 0.31798 | 1.2 |
| FABPH_13_25 | 2.666667 | 1 | 3.785939 | 1.419727 | 2.666667 |
| FAK1_569_581 | 21.66667 | 23 | 3.21455 | 0.148364 | 0.942029 |
| FAK2_572_584 | 40 | 42 | 4.358899 | 0.108972 | 0.952381 |
| FER_707_719 | 50 | 49 | 3.605551 | 0.072111 | 1.020408 |
| FES_706_718 | 72.66667 | 70 | 8.326664 | 0.114587 | 1.038095 |
| FGFR1_761_773 | 4.333333 | 4 | 4.50925 | 1.040596 | 1.083333 |
| FGFR2_762_774 | 27 | 22 | 13.22876 | 0.489954 | 1.227273 |
| FGFR3_641_653 | 3 | 2 | 3.605551 | 1.20185 | 1.5 |
| FGFR3_753_765 | 18.66667 | 18 | 6.027714 | 0.322913 | 1.037037 |
| FRK_380_392 | 237 | 240 | 14.73092 | 0.062156 | 0.9875 |
| INSR_1348_1360 | 14 | 13 | 3.605551 | 0.257539 | 1.076923 |
| INSR_992_1004 | 12.33333 | 12 | 10.50397 | 0.851673 | 1.027778 |
| JAK1_1015_1027 | 22.33333 | 21 | 9.073772 | 0.406288 | 1.063492 |
| JAK2_563_577 | 39.66667 | 34 | 12.50333 | 0.31521 | 1.166667 |
| K2C6B_53_65 | 55.66667 | 57 | 9.073772 | 0.163002 | 0.976608 |
| K2C8_425_437 | 14.66667 | 14 | 6.027714 | 0.41098 | 1.047619 |
| KSYK_518_530 | 24.66667 | 28 | 11.37248 | 0.461047 | 0.880952 |
| LAT_194_206 | 10 | 10 | 9 | 0.9 | 1 |
| LAT_249_261 | 54.66667 | 47 | 14.15392 | 0.258913 | 1.163121 |
| LCK_387_399 | 30.66667 | 30 | 5.033223 | 0.164127 | 1.022222 |
| MBP_198_210 | 33.33333 | 33 | 0.57735 | 0.017321 | 1.010101 |
| MBP_259_271 | 12.33333 | 10 | 6.806859 | 0.551908 | 1.233333 |
| MBP_263_275 | 9 | 8 | 4.582576 | 0.509175 | 1.125 |
| MET_1227_1239 | 32.66667 | 33 | 5.507571 | 0.168599 | 0.989899 |
| MK01_180_192 | 19.66667 | 18 | 3.785939 | 0.192505 | 1.092593 |
| MK01_198_210 | 2 | 0 | 3.464102 | 1.732051 | Inf |
| MK07_211_223 | 4.333333 | 2 | 4.932883 | 1.138358 | 2.166667 |
| MK10_216_228 | 60.33333 | 59 | 13.05118 | 0.216318 | 1.022599 |
| MK12_178_190 | 10 | 7 | 7 | 0.7 | 1.428571 |
| MK14_173_185 | 0 | 0 | 0 | NA | NA |
| NCF1_313_325 | 64.33333 | 60 | 10.21437 | 0.158773 | 1.072222 |
| NPT2A_501_513 | 7.333333 | 6 | 8.082904 | 1.102214 | 1.222222 |
| NTRK1_489_501 | 11.66667 | 11 | 9.0185 | 0.773014 | 1.060606 |
| NTRK2_509_521 | 0 | 0 | 0 | NA | NA |
| NTRK2_696_708 | 48.66667 | 46 | 5.507571 | 0.113169 | 1.057971 |
| ODBA_340_352 | 13 | 7 | 10.3923 | 0.799408 | 1.857143 |
| ODPAT_291_303 | 9.333333 | 10 | 1.154701 | 0.123718 | 0.933333 |
| PP2AB_297_309 | 34 | 30 | 7.81025 | 0.229713 | 1.133333 |
| P85A_600_612 | 108 | 101 | 16.64332 | 0.154105 | 1.069307 |
| PAXI_111_123 | 165 | 174 | 15.58846 | 0.094475 | 0.948276 |
| PAXI_24_36 | 112.6667 | 121 | 15.30795 | 0.135869 | 0.931129 |
| PDPK1_2_14 | 57.33333 | 60 | 12.2202 | 0.213143 | 0.955556 |
| PDPK1_369_381 | 44.66667 | 39 | 10.69268 | 0.239388 | 1.145299 |
| PECA1_706_718 | 69 | 71 | 5.291503 | 0.076688 | 0.971831 |
| PERI_458_470 | 1 | 0 | 1.732051 | 1.732051 | Inf |
| PGFRB_1002_1014 | 18 | 13 | 8.660254 | 0.481125 | 1.384615 |
| PGFRB_572_584 | 141 | 139 | 11.13553 | 0.078975 | 1.014388 |
| PGFRB_709_721 | 24.33333 | 24 | 3.511885 | 0.144324 | 1.013889 |
| PGFRB_768_780 | 19.66667 | 18 | 5.686241 | 0.289131 | 1.092593 |
| PGFRB_771_783 | 29 | 23 | 11.26943 | 0.388601 | 1.26087 |
| PLCG1_1246_1258 | 11 | 8 | 6.082763 | 0.552978 | 1.375 |
| PLCG1_764_776 | 262.6667 | 277 | 39.50105 | 0.150385 | 0.948255 |
| PLCG1_776_788 | 3.333333 | 4 | 3.05505 | 0.916515 | 0.833333 |
| PRGR_545_557 | 2.333333 | 0 | 4.041452 | 1.732051 | Inf |
| PRGR_786_798 | 2.666667 | 0 | 4.618802 | 1.732051 | Inf |
| PRRX2_202_214 | 26 | 26 | 8 | 0.307692 | 1 |
| PTN11_539_551 | 18.33333 | 19 | 6.027714 | 0.328784 | 0.964912 |
| RAF1_332_344 | 82.66667 | 82 | 8.020806 | 0.097026 | 1.00813 |
| RASA1_453_465 | 38 | 37 | 3.605551 | 0.094883 | 1.027027 |
| RB_804_816 | 30 | 28 | 10.14889 | 0.338296 | 1.071429 |
| RBL2_99_111 | 1 | 1 | 1 | 1 | 1 |
| RET_1022_1034 | 105.3333 | 96 | 17.92577 | 0.170181 | 1.097222 |
| RET_680_692 | 2.333333 | 0 | 4.041452 | 1.732051 | Inf |
| RON_1346_1358 | 22.66667 | 19 | 7.234178 | 0.319155 | 1.192982 |
| RON_1353_1365 | 19.33333 | 18 | 5.131601 | 0.265428 | 1.074074 |
| SRC8_CHICK_470_482 | 5.333333 | 0 | 9.237604 | 1.732051 | Inf |
| SRC8_CHICK_476_488 | 372.3333 | 376 | 27.68273 | 0.074349 | 0.990248 |
| SRC8_CHICK_492_504 | 486.6667 | 477 | 25.8908 | 0.0532 | 1.020266 |
| STA5A_687_699 | 0 | 0 | 0 | NA | NA |
| STAT1_694_706 | 0.666667 | 0 | 1.154701 | 1.732051 | Inf |
| STAT3_698_710 | 5 | 2 | 7 | 1.4 | 2.5 |
| STAT4_686_698 | 16.33333 | 14 | 6.806859 | 0.416746 | 1.166667 |
| STAT4_714_726 | 9 | 5 | 9.643651 | 1.071517 | 1.8 |
| STAT6_634_646 | 3.333333 | 0 | 5.773503 | 1.732051 | Inf |
| TEC_512_524 | 28 | 24 | 10.58301 | 0.377964 | 1.166667 |
| TNNT1_2_14 | 4.333333 | 0 | 7.505553 | 1.732051 | Inf |
| TYRO3_679_691 | 39 | 38 | 10.53565 | 0.270145 | 1.026316 |
| VGFR1_1040_1052 | 7 | 5 | 7.211103 | 1.030158 | 1.4 |
| VGFR1_1046_1058_Y1048F | 2.666667 | 2 | 3.05505 | 1.145644 | 1.333333 |
| VGFR1_1049_1061 | 7.333333 | 9 | 5.686241 | 0.775396 | 0.814815 |
| VGFR1_1162_1174 | 0.333333 | 0 | 0.57735 | 1.732051 | Inf |
| VGFR1_1206_1218 | 3 | 1 | 4.358899 | 1.452966 | 3 |
| VGFR1_1235_1247 | 0 | 0 | 0 | NA | NA |
| VGFR1_1320_1332_C1320S/C1321S | 5.666667 | 5 | 4.041452 | 0.713197 | 1.133333 |
| VGFR1_1326_1338 | 22.33333 | 19 | 10.40833 | 0.466045 | 1.175439 |
| VGFR2_1046_1058 | 26.66667 | 24 | 11.23981 | 0.421493 | 1.111111 |
| VGFR2_1052_1064 | 20.66667 | 18 | 9.291573 | 0.449592 | 1.148148 |
| VGFR2_1168_1180 | 6.333333 | 7 | 3.05505 | 0.482376 | 0.904762 |
| VGFR2_1207_1219_C1208S | 0.333333 | 0 | 0.57735 | 1.732051 | Inf |
| VGFR2_944_956 | 42 | 43 | 6.557439 | 0.156129 | 0.976744 |
| VGFR2_989_1001 | 75.33333 | 70 | 11.93035 | 0.158368 | 1.07619 |
| VGFR3_1061_1073 | 7 | 8 | 5.567764 | 0.795395 | 0.875 |
| VINC_815_827 | 21 | 19 | 5.291503 | 0.251976 | 1.105263 |
| ZAP70_485_497 | 27.66667 | 26 | 4.725816 | 0.170813 | 1.064103 |
| ZBT16_621_633 | 16.33333 | 17 | 4.041452 | 0.247436 | 0.960784 |

# Stats from nanoHA group

Table 8 - Summary of the statistical parameters of each spot for nanoHA group.

| spot | A | M | SD | P1 | P2 |
| --- | --- | --- | --- | --- | --- |
| 41_654_666 | 68.66667 | 71 | 22.59056 | 0.328989 | 0.967136 |
| ACHD_383_395 | 5.333333 | 0 | 9.237604 | 1.732051 | Inf |
| AMPE_5_17 | 4.666667 | 6 | 4.163332 | 0.892143 | 0.777778 |
| ANXA1_14_26 | 30 | 33 | 12.76715 | 0.425572 | 0.909091 |
| ANXA2_17_29 | 3 | 0 | 5.196152 | 1.732051 | Inf |
| ART_004_EAIYAAPFAKKKXC | 359.3333 | 326 | 59.47549 | 0.165516 | 1.102249 |
| B3AT_39_51 | 0 | 0 | 0 | NA | NA |
| CTNB1_79_91 | 11.33333 | 15 | 10.01665 | 0.883822 | 0.755556 |
| C1R_199_211 | 6.666667 | 3 | 9.073772 | 1.361066 | 2.222222 |
| CALM_93_105 | 0.666667 | 0 | 1.154701 | 1.732051 | Inf |
| PGFRB_1014_1028 | 25.33333 | 24 | 26.02563 | 1.027327 | 1.055556 |
| ART_003_EAI(pY)AAPFAKKKXC | 1384.333 | 1339 | 510.5118 | 0.368778 | 1.033856 |
| CALM_95_107 | 6 | 1 | 9.539392 | 1.589899 | 6 |
| CBL_693_705 | 21.66667 | 9 | 25.48202 | 1.176093 | 2.407407 |
| CD3Z_116_128 | 4 | 0 | 6.928203 | 1.732051 | Inf |
| CD3Z_146_158 | 0.666667 | 0 | 1.154701 | 1.732051 | Inf |
| CD79A_181_193 | 1271.333 | 1292 | 225.7107 | 0.177539 | 0.984004 |
| CDK2_8_20 | 133.3333 | 142 | 18.58315 | 0.139374 | 0.938967 |
| CDK7_157_169 | 28.66667 | 25 | 10.01665 | 0.349418 | 1.146667 |
| CRK_214_226 | 14.66667 | 13 | 8.621678 | 0.587842 | 1.128205 |
| DCX_109_121 | 58.66667 | 53 | 15.30795 | 0.260931 | 1.106918 |
| DDR1_506_518 | 4.666667 | 0 | 8.082904 | 1.732051 | Inf |
| DYR1A_212_224 | 0.333333 | 0 | 0.57735 | 1.732051 | Inf |
| DYR1A_312_324 | 12.66667 | 5 | 15.04438 | 1.187714 | 2.533333 |
| EFS_246_258 | 483 | 471 | 126.4278 | 0.261755 | 1.025478 |
| EFS_246_258_Y253F | 1.333333 | 0 | 2.309401 | 1.732051 | Inf |
| EGFR_1062_1074 | 3.333333 | 0 | 5.773503 | 1.732051 | Inf |
| EGFR_1103_1115 | 12.66667 | 9 | 6.350853 | 0.501383 | 1.407407 |
| EGFR_1118_1130 | 1.333333 | 0 | 2.309401 | 1.732051 | Inf |
| EGFR_1165_1177 | 35.66667 | 33 | 9.291573 | 0.260511 | 1.080808 |
| EGFR_1190_1202 | 1.666667 | 0 | 2.886751 | 1.732051 | Inf |
| EGFR_862_874 | 2.333333 | 0 | 4.041452 | 1.732051 | Inf |
| EGFR_908_920 | 3.333333 | 0 | 5.773503 | 1.732051 | Inf |
| ENOG_37_49 | 438.3333 | 443 | 76.10738 | 0.173629 | 0.989466 |
| EPHA1_774_786 | 120.3333 | 113 | 24.82606 | 0.206311 | 1.064897 |
| EPHA2_581_593 | 4 | 3 | 4.582576 | 1.145644 | 1.333333 |
| EPHA2_765_777 | 120.3333 | 103 | 40.85748 | 0.339536 | 1.168285 |
| EPHA4_589_601 | 19.33333 | 16 | 13.31666 | 0.688793 | 1.208333 |
| EPHA4_921_933 | 4.333333 | 4 | 4.50925 | 1.040596 | 1.083333 |
| EPHA7_607_619 | 53.66667 | 47 | 18.90326 | 0.352235 | 1.141844 |
| EPHB1_771_783 | 40.33333 | 33 | 20.03331 | 0.496694 | 1.222222 |
| EPHB1_921_933 | 41.66667 | 31 | 22.03028 | 0.528727 | 1.344086 |
| EPHB4_583_595 | 6.333333 | 7 | 4.041452 | 0.638124 | 0.904762 |
| EPOR_361_373 | 25.66667 | 23 | 17.15615 | 0.668421 | 1.115942 |
| EPOR_419_431 | 34.66667 | 25 | 16.74316 | 0.482976 | 1.386667 |
| ERBB2_1241_1253 | 23.66667 | 20 | 17.78576 | 0.751511 | 1.183333 |
| ERBB2_870_882 | 16 | 11 | 15.13275 | 0.945797 | 1.454545 |
| ERBB4_1181_1193 | 3.333333 | 0 | 5.773503 | 1.732051 | Inf |
| ERBB4_1277_1289 | 23.33333 | 20 | 21.19748 | 0.908464 | 1.166667 |
| FABPH_13_25 | 1.333333 | 0 | 2.309401 | 1.732051 | Inf |
| FAK1_569_581 | 13.33333 | 11 | 5.859465 | 0.43946 | 1.212121 |
| FAK2_572_584 | 38.33333 | 35 | 11.37248 | 0.296673 | 1.095238 |
| FER_707_719 | 42.66667 | 37 | 11.59023 | 0.271646 | 1.153153 |
| FES_706_718 | 81.33333 | 74 | 12.70171 | 0.156169 | 1.099099 |
| FGFR1_761_773 | 2.333333 | 0 | 4.041452 | 1.732051 | Inf |
| FGFR2_762_774 | 19.33333 | 16 | 14.29452 | 0.739372 | 1.208333 |
| FGFR3_641_653 | 3 | 0 | 5.196152 | 1.732051 | Inf |
| FGFR3_753_765 | 20.66667 | 16 | 9.865766 | 0.477376 | 1.291667 |
| FRK_380_392 | 260.3333 | 259 | 31.0215 | 0.119161 | 1.005148 |
| INSR_1348_1360 | 7.666667 | 6 | 7.637626 | 0.996212 | 1.277778 |
| INSR_992_1004 | 9.666667 | 4 | 13.42882 | 1.389189 | 2.416667 |
| JAK1_1015_1027 | 16.66667 | 13 | 10.01665 | 0.600999 | 1.282051 |
| JAK2_563_577 | 38.33333 | 30 | 15.30795 | 0.399338 | 1.277778 |
| K2C6B_53_65 | 44.66667 | 37 | 14.15392 | 0.316879 | 1.207207 |
| K2C8_425_437 | 6.333333 | 6 | 6.506407 | 1.027327 | 1.055556 |
| KSYK_518_530 | 16.33333 | 16 | 8.504901 | 0.520708 | 1.020833 |
| LAT_194_206 | 10.66667 | 6 | 13.61372 | 1.276286 | 1.777778 |
| LAT_249_261 | 43.66667 | 40 | 17.78576 | 0.407308 | 1.091667 |
| LCK_387_399 | 25.66667 | 22 | 12.89703 | 0.502482 | 1.166667 |
| MBP_198_210 | 26.33333 | 25 | 8.082904 | 0.306946 | 1.053333 |
| MBP_259_271 | 6.666667 | 7 | 6.506407 | 0.975961 | 0.952381 |
| MBP_263_275 | 5.333333 | 3 | 5.859465 | 1.09865 | 1.777778 |
| MET_1227_1239 | 35.66667 | 36 | 4.50925 | 0.126428 | 0.990741 |
| MK01_180_192 | 16.33333 | 11 | 10.11599 | 0.619347 | 1.484848 |
| MK01_198_210 | 0.333333 | 0 | 0.57735 | 1.732051 | Inf |
| MK07_211_223 | 2.333333 | 0 | 4.041452 | 1.732051 | Inf |
| MK10_216_228 | 47 | 45 | 14.10674 | 0.300143 | 1.044444 |
| MK12_178_190 | 7.666667 | 8 | 7.505553 | 0.978985 | 0.958333 |
| MK14_173_185 | 2 | 0 | 3.464102 | 1.732051 | Inf |
| NCF1_313_325 | 55 | 52 | 5.196152 | 0.094475 | 1.057692 |
| NPT2A_501_513 | 5.666667 | 2 | 8.144528 | 1.43727 | 2.833333 |
| NTRK1_489_501 | 7.333333 | 8 | 5.033223 | 0.686349 | 0.916667 |
| NTRK2_509_521 | 0 | 0 | 0 | NA | NA |
| NTRK2_696_708 | 39 | 44 | 8.660254 | 0.222058 | 0.886364 |
| ODBA_340_352 | 9.666667 | 3 | 14.22439 | 1.471489 | 3.222222 |
| ODPAT_291_303 | 5.666667 | 5 | 6.027714 | 1.063714 | 1.133333 |
| PP2AB_297_309 | 30.33333 | 31 | 11.01514 | 0.363137 | 0.978495 |
| P85A_600_612 | 110.6667 | 92 | 34.07834 | 0.307937 | 1.202899 |
| PAXI_111_123 | 173.6667 | 169 | 38.21431 | 0.220044 | 1.027613 |
| PAXI_24_36 | 111.6667 | 106 | 18.17507 | 0.162762 | 1.053459 |
| PDPK1_2_14 | 53.66667 | 47 | 14.22439 | 0.265051 | 1.141844 |
| PDPK1_369_381 | 46 | 39 | 15.71623 | 0.341657 | 1.179487 |
| PECA1_706_718 | 71.66667 | 75 | 7.571878 | 0.105654 | 0.955556 |
| PERI_458_470 | 1.666667 | 0 | 2.886751 | 1.732051 | Inf |
| PGFRB_1002_1014 | 14.66667 | 7 | 19.65536 | 1.340138 | 2.095238 |
| PGFRB_572_584 | 138.3333 | 122 | 35.44479 | 0.256227 | 1.13388 |
| PGFRB_709_721 | 22.66667 | 18 | 15.53491 | 0.685364 | 1.259259 |
| PGFRB_768_780 | 17 | 13 | 7.81025 | 0.459426 | 1.307692 |
| PGFRB_771_783 | 26.33333 | 22 | 13.05118 | 0.495614 | 1.19697 |
| PLCG1_1246_1258 | 9 | 9 | 9 | 1 | 1 |
| PLCG1_764_776 | 267.3333 | 249 | 48.19059 | 0.180264 | 1.073628 |
| PLCG1_776_788 | 2 | 0 | 3.464102 | 1.732051 | Inf |
| PRGR_545_557 | 2 | 0 | 3.464102 | 1.732051 | Inf |
| PRGR_786_798 | 1 | 0 | 1.732051 | 1.732051 | Inf |
| PRRX2_202_214 | 28.33333 | 29 | 6.027714 | 0.212743 | 0.977011 |
| PTN11_539_551 | 16 | 17 | 8.544004 | 0.534 | 0.941176 |
| RAF1_332_344 | 80 | 79 | 6.557439 | 0.081968 | 1.012658 |
| RASA1_453_465 | 32 | 32 | 11 | 0.34375 | 1 |
| RB_804_816 | 22.66667 | 17 | 17.21434 | 0.759456 | 1.333333 |
| RBL2_99_111 | 3 | 0 | 5.196152 | 1.732051 | Inf |
| RET_1022_1034 | 112 | 99 | 23.38803 | 0.208822 | 1.131313 |
| RET_680_692 | 3.333333 | 0 | 5.773503 | 1.732051 | Inf |
| RON_1346_1358 | 13 | 9 | 15.3948 | 1.184216 | 1.444444 |
| RON_1353_1365 | 11 | 7 | 13.45362 | 1.223057 | 1.571429 |
| SRC8_CHICK_470_482 | 4 | 0 | 6.928203 | 1.732051 | Inf |
| SRC8_CHICK_476_488 | 383.6667 | 383 | 44.00379 | 0.114693 | 1.001741 |
| SRC8_CHICK_492_504 | 521 | 524 | 65.55151 | 0.125819 | 0.994275 |
| STA5A_687_699 | 2.333333 | 0 | 4.041452 | 1.732051 | Inf |
| STAT1_694_706 | 1.333333 | 0 | 2.309401 | 1.732051 | Inf |
| STAT3_698_710 | 5 | 1 | 7.81025 | 1.56205 | 5 |
| STAT4_686_698 | 6.333333 | 0 | 10.96966 | 1.732051 | Inf |
| STAT4_714_726 | 10 | 6 | 12.49 | 1.249 | 1.666667 |
| STAT6_634_646 | 2.666667 | 2 | 3.05505 | 1.145644 | 1.333333 |
| TEC_512_524 | 22.66667 | 15 | 14.15392 | 0.624437 | 1.511111 |
| TNNT1_2_14 | 4.666667 | 0 | 8.082904 | 1.732051 | Inf |
| TYRO3_679_691 | 36 | 36 | 4 | 0.111111 | 1 |
| VGFR1_1040_1052 | 10 | 6 | 12.49 | 1.249 | 1.666667 |
| VGFR1_1046_1058_Y1048F | 4.333333 | 0 | 7.505553 | 1.732051 | Inf |
| VGFR1_1049_1061 | 3.333333 | 3 | 3.511885 | 1.053565 | 1.111111 |
| VGFR1_1162_1174 | 2.333333 | 0 | 4.041452 | 1.732051 | Inf |
| VGFR1_1206_1218 | 2.666667 | 0 | 4.618802 | 1.732051 | Inf |
| VGFR1_1235_1247 | 0.333333 | 0 | 0.57735 | 1.732051 | Inf |
| VGFR1_1320_1332_C1320S/C1321S | 5 | 0 | 8.660254 | 1.732051 | Inf |
| VGFR1_1326_1338 | 27 | 21 | 11.26943 | 0.417386 | 1.285714 |
| VGFR2_1046_1058 | 17 | 12 | 13.22876 | 0.778162 | 1.416667 |
| VGFR2_1052_1064 | 15 | 9 | 13.0767 | 0.87178 | 1.666667 |
| VGFR2_1168_1180 | 5 | 0 | 8.660254 | 1.732051 | Inf |
| VGFR2_1207_1219_C1208S | 1.333333 | 0 | 2.309401 | 1.732051 | Inf |
| VGFR2_944_956 | 32.33333 | 35 | 4.618802 | 0.14285 | 0.92381 |
| VGFR2_989_1001 | 71.33333 | 73 | 7.637626 | 0.10707 | 0.977169 |
| VGFR3_1061_1073 | 4.666667 | 5 | 3.511885 | 0.752547 | 0.933333 |
| VINC_815_827 | 15.33333 | 14 | 5.131601 | 0.33467 | 1.095238 |
| ZAP70_485_497 | 22 | 18 | 9.643651 | 0.438348 | 1.222222 |
| ZBT16_621_633 | 8.666667 | 12 | 7.571878 | 0.873678 | 0.722222 |
